# Neurospora Importin α Is Required for Normal Heterochromatic Formation and DNA Methylation

**DOI:** 10.1371/journal.pgen.1005083

**Published:** 2015-03-20

**Authors:** Andrew D. Klocko, Michael R. Rountree, Paula L. Grisafi, Shan M. Hays, Keyur K. Adhvaryu, Eric U. Selker

**Affiliations:** Institute of Molecular Biology, University of Oregon, Eugene, Oregon, United States of America; Centro de Investigaciones Biológicas, Spain

## Abstract

Heterochromatin and associated gene silencing processes play roles in development, genome defense, and chromosome function. In many species, constitutive heterochromatin is decorated with histone H3 tri-methylated at lysine 9 (H3K9me3) and cytosine methylation. In *Neurospora crassa*, a five-protein complex, DCDC, catalyzes H3K9 methylation, which then directs DNA methylation. Here, we identify and characterize a gene important for DCDC function, *dim-3* (*d*efective *i*n *m*ethylation-3), which encodes the nuclear import chaperone NUP-6 (Importin α). The critical mutation in *dim-3* results in a substitution in an ARM repeat of NUP-6 and causes a substantial loss of H3K9me3 and DNA methylation. Surprisingly, nuclear transport of all known proteins involved in histone and DNA methylation, as well as a canonical transport substrate, appear normal in *dim-3* strains. Interactions between DCDC members also appear normal, but the *nup-6dim-3* allele causes the DCDC members DIM-5 and DIM-7 to mislocalize from heterochromatin and NUP-6^*dim-3*^ itself is mislocalized from the nuclear envelope, at least in conidia. GCN-5, a member of the SAGA histone acetyltransferase complex, also shows altered localization in *dim-3*, raising the possibility that NUP-6 is necessary to localize multiple chromatin complexes following nucleocytoplasmic transport.

## Author Summary

The epigenetic information contained in chromatin is essential for development of higher organisms, and if misregulated, can lead to the unregulated growth associated with human cancers. Chromatin is typically classified into two basic types: gene-rich 'euchromatin', and gene-poor heterochromatin, which is also rich in repeated DNA and 'repressive chromatin marks'. As in humans and eukaryotes generally, heterochromatin in *Neurospora crassa* is decorated with DNA methylation and histone H3 lysine 9 (H3K9) methylation, but unlike the case in mammals, loss of these epigenetic marks does not compromise viability. In Neurospora, the DCDC, a five-member Cul4-based protein complex, trimethylates H3K9. Little information is available on the regulation of DCDC or similar complexes in other organisms. Using forward genetics, we identified a novel role for Importin α (NUP-6) for the function of DCDC. Although NUP-6 typically functions in nucleocytoplasmic transport, the *dim-3* strain, which contains an altered *nup-6* gene that reduces DNA methylation and H3K9me3, shows normal nuclear transport of the heterochromatin machinery and a canonical transport substrate. Two DCDC members are mislocalized from heterochromatin in the *dim-3* mutant, signifying that NUP-6 may be important for targeting key proteins to incipient heterochromatic DNA. The euchromatic complex SAGA has increased euchromatin localization in *dim-3*, suggesting that NUP-6 may localize multiple chromatin complexes to sub-nuclear genomic targets.

## Introduction

The densely staining regions of eukaryotic chromosomes, referred to as heterochromatin, typically contain repetitive, A:T rich DNA, and are characterized by low gene density, reduced genetic recombination, di- or tri-methylation of lysine 9 on histone H3 (H3K9me2 or H3K9me3), and DNA methylation [1–4]. Heterochromatin is critical for centromere and telomere function, and is largely responsible for the silencing of transposable elements [1,4]. Unlike the situation in animals and plants, which require DNA methylation for normal development [5,6], the fungus *Neurospora crassa* does not require DNA methylation for viability [4,7]. Neurospora has characteristics of heterochromatin found in higher eukaryotes and is convenient for genetic and biochemical studies. These traits have led to the identification of genes involved in establishing, maintaining, and regulating DNA methylation and other features of heterochromatin [8]

A single DNA methyltransferase (DNMTase), DIM-2, is responsible for all DNA methylation in vegetative tissue of Neurospora [9]. DIM-2 directly interacts with heterochromatin protein-1 (HP1) [10], which binds to H3K9me3 [11,12]. The histone methyltransferase (HMTase) DIM-5 [13,14] is responsible for trimethylation of H3K9. *In vivo*, DIM-5 activity depends on all members of the five protein complex, DCDC (DIM-5/-7/-9, CUL4, DDB1 Complex) but DIM-7 alone appears sufficient to target DIM-5 to incipient heterochromatin regions [8,15]. DCDC resembles Cullin-4 E3 ubiquitin ligase complexes, with the WD-40 protein DIM-9 being the putative DCAF (DDB1/CUL4 associated factor), which is normally expected to recognize substrates destined for ubiquitination. However, results of recent studies indicate that DCDC does not function as a canonical ubiquitin ligase [16]. Thus, important questions regarding how DCDC and other members of the heterochromatin/DNA methylation machinery function and are controlled remain unanswered.

To improve our understanding of the control of DNA methylation and heterochromatin formation, we characterized the Neurospora *dim-3* strain, which shows a substantial loss of DNA methylation [7]. Genetic mapping, whole genome sequencing, and complementation tests identified the causative mutation in the *nup-6* gene, resulting in a critical change in the eighth ARM repeat of NUP-6 (Importin α). This protein (also known as Srp1p in yeast and karyopherin α in humans) is the canonical nucleocytoplasmic transport adaptor. Importin α binds “cargo” proteins to be transported into the nucleus, complexes with Importin β, and then is shuttled through the nuclear pore complex to the nucleoplasm [17–19]. We found that *dim-3* strains have a drastic reduction in global H3K9me3, indicating that NUP-6 is required for proper DCDC function. Nuclear transport and interactions of critical DCDC components appear normal in *dim-3* strains but at least two DCDC components (DIM-5 and DIM-7) are mislocalized from heterochromatin. Curiously, the SAGA histone acetyltransferase, but not DCDC components, showed increased localization in euchromatin of a *dim-3* strain. Altogether, our results reveal a nuclear transport-independent role of NUP-6 in localizing chromatin complexes to sub-nuclear targets.

## Results

### Loss of DNA methylation in *dim-3* strains is caused by a mutation in the *nup-6* gene

**Fig 1:**
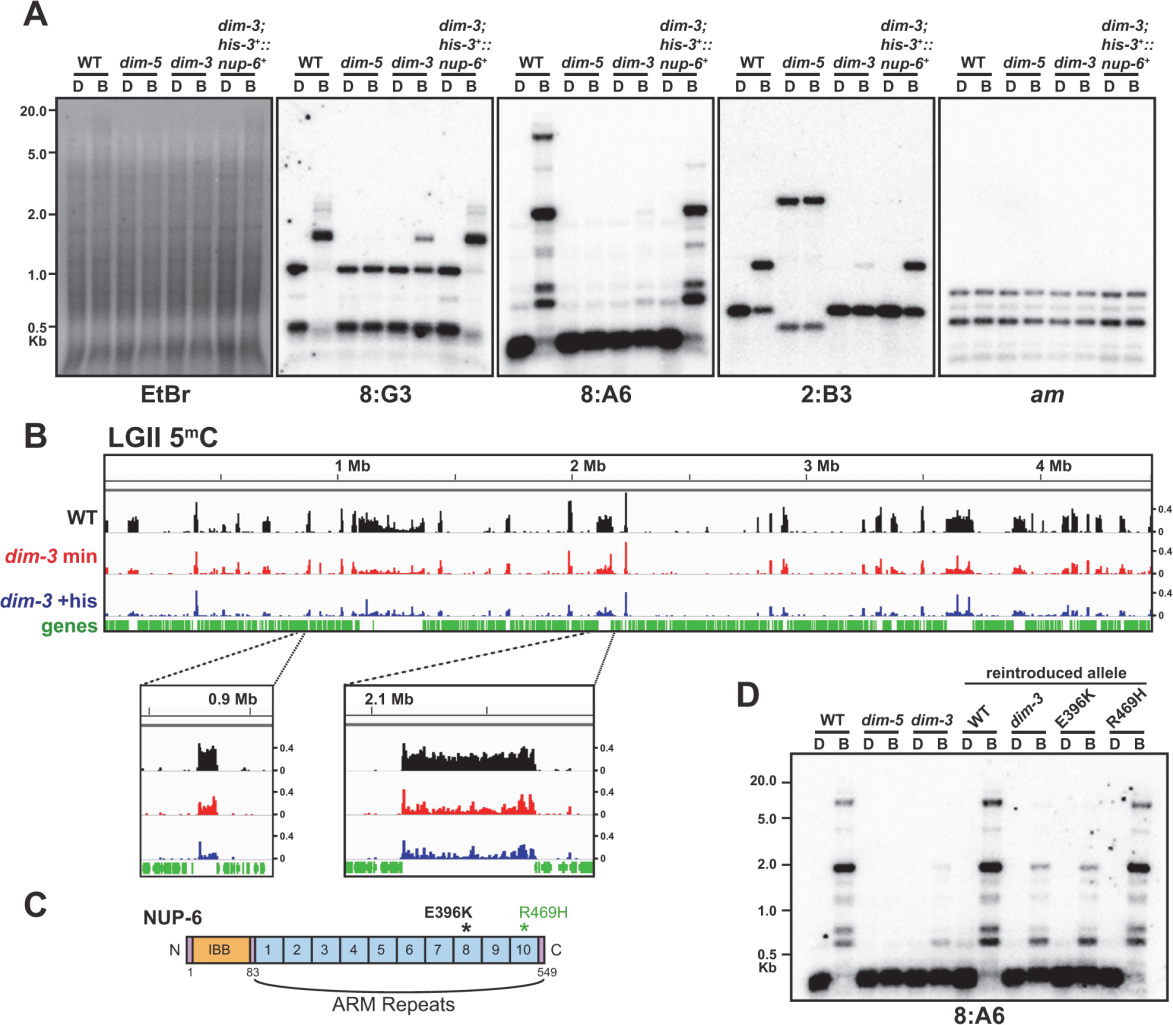
The DNA methylation deficiency in *dim-3* strains is caused by mutations in *nup-6*. [A]. Southern blots showing DNA methylation loss in a *dim-3* strain and complementation by *nup-6* ^*+*^ at the *his-3* locus. The ectopic *his-3* ^*+*^::*nup-6* ^*+*^ strain was grown in the presence of histidine for comparison with the auxotrophic *dim-3* strain. Genomic DNA from *dim-3* ^*+*^ (Wild type [“WT”]), *dim-5*, *dim-3; his-3*, and *dim-3; his-3* ^*+*^::*nup-6* ^*+*^ strains was digested with the 5^m^C-insensitive restriction endonuclease DpnII (D) or its 5^m^C-sensitive isoschizomer BfuCI (B), fractionated on an agarose gel, transferred to a nylon membrane, and probed for heterochromatic regions (8:A6, 8:G3, and 2:B3; [20]) or an unmethylated region (*am*). The ethidium bromide (EtBr)-stained gel, with the positions of size markers (Kb) indicated, is shown because it provides an indication of global differences in DNA methylation. A reported restriction site polymorphism in the 2:B3 region is evident for the *dim-5* strain [11]. [B] Bisulfite sequencing (BS-seq) of methylated cytosines from *dim-3* ^+^ (Wild type, “WT”, black track) and *dim-3* strains (grown in minimal medium = red track, grown in medium containing histidine = blue track) displayed by the Integrative Genomics Viewer, with y-axis denoting the number of normalized mapped reads (reads*10^6^/total number of mapped reads; [58]). Linkage Group II (LGII) is shown, and two heterochromatic peaks are displayed at higher magnification below. Genes are displayed on the x-axis below the 5^m^C peaks as vertical lines, while distance (in Megabases) from the left end of LG II is displayed above the graph. Due to the reduced cytosine methylation in the *dim-3* samples, the signal-to-noise ratio is lower, which results in disproportionally high background peaks. [C] Schematic of NUP-6 structure, with ARM repeats 1–10, the N-terminal Importin β binding domain (IBB), and the mutations found in the *dim-3* allele indicated (penetrant mutation, E396K, is shown in black). [D] Southern blot assay of indicated mutations reintroduced into a *dim-3* ^+^ (WT) strain, along with *dim-5*, *dim-3*, and WT controls.

The *dim-3* gene was genetically identified in a brute-force screen for methylation defects following N-methyl-N’-nitro-N-nitrosoguanidine mutagenesis [7]. Southern blots probed for the representative interspersed heterochromatic regions 8:G3, 8:A6, and 2:B3 [20] in a histidine auxotrophic *dim-3* strain illustrate the substantial DNA methylation reduction caused by the allele (Fig. 1A). Genome-wide bisulfite sequencing (BS-Seq; Rountree and Selker, in preparation) demonstrated that the residual DNA methylation in a *dim-3* mutant is distributed normally, or largely normally, to heterochromatic regions (Figs. 1B and S1).

*Dim-3* was mapped to the right arm of Linkage Group V, and high-throughput sequencing identified two point mutations in the open reading frame of gene NCU01249, E396K and R469H; both were confirmed by Sanger sequencing. NCU01249 encodes NUP-6 (NIH GenBank accession EAA31416.1), which is predicted to form a structure similar to yeast Importin α [21], a protein that is highly conserved in eukaryotes [19]. Neurospora NUP-6 includes an N-terminal ∼80 amino acid Importin β-binding domain (IBB), followed by ten ∼40 amino acid ARM repeats (Fig. 1C), each of which should fold into a triple-alpha helical bundle to form a binding pocket for the nuclear localization signals (NLS) of cargo proteins (S2A Fig.; [22]). ARM repeats 1–9 are thought to recognize the NLS [22] while cargo release factors interact with the less-conserved 10^th^ ARM repeat [23]. The E396K and R469H changes in *nup-6* ^*dim-3*^ are in the putative 8^th^ and 10^th^ ARM repeats, respectively (Figs. 1C and S2A), and should not destabilize the NUP-6^*dim-3*^ protein nor impact its nuclear shuttling (S2B-S2C Fig.). Interestingly, *dim-3* strains have a minor growth defect (S2D Fig.) and are homozygous sterile, indicating that one or both of the amino acid substitutions compromise an important cellular process.

To confirm that *nup-6* ^*dim-3*^ causes the observed DNA methylation loss in *dim-3* strains, we introduced a wild type (WT) *nup-6* gene (*nup-6* ^*+*^) at an ectopic locus (*his-3*) of a *dim-3* strain. Ectopic *nup-6* ^*+*^ restored global DNA methylation to WT levels at all the representative heterochromatic loci tested (Fig. 1A), suggesting that *nup-6* ^*dim-3*^ was indeed responsible for the Dim phenotype. To determine if one or both of the mutations cause the phenotype, we replaced the endogenous *nup-6* ^*+*^ allele with engineered mutant alleles. As expected, the strain with the reintroduced *nup-6* ^*dim-3*^ allele showed reduced DNA methylation, although somewhat less so than the original *dim-3* isolate (Figs. 1D and S3). Reintroduction of the R469H mutation did not affect DNA methylation, whereas the reintroduced E396K mutation caused a loss equivalent to that observed with the reintroduced *nup-6* ^*dim-3*^ allele, implicating this change as the causative mutation (Figs. 1D and S3). As expected, the *nup-6* gene appears essential, because strains with the gene deleted are only viable as heterokaryons, containing both *nup-6* ^*+*^ and Δ*nup-6*::*hph* nuclei [24]. Thus, there is no reason to expect that the E396K creates a null allele.

### Histidine exacerbates the *dim-3*-mediated DNA methylation loss

**Fig 2:**
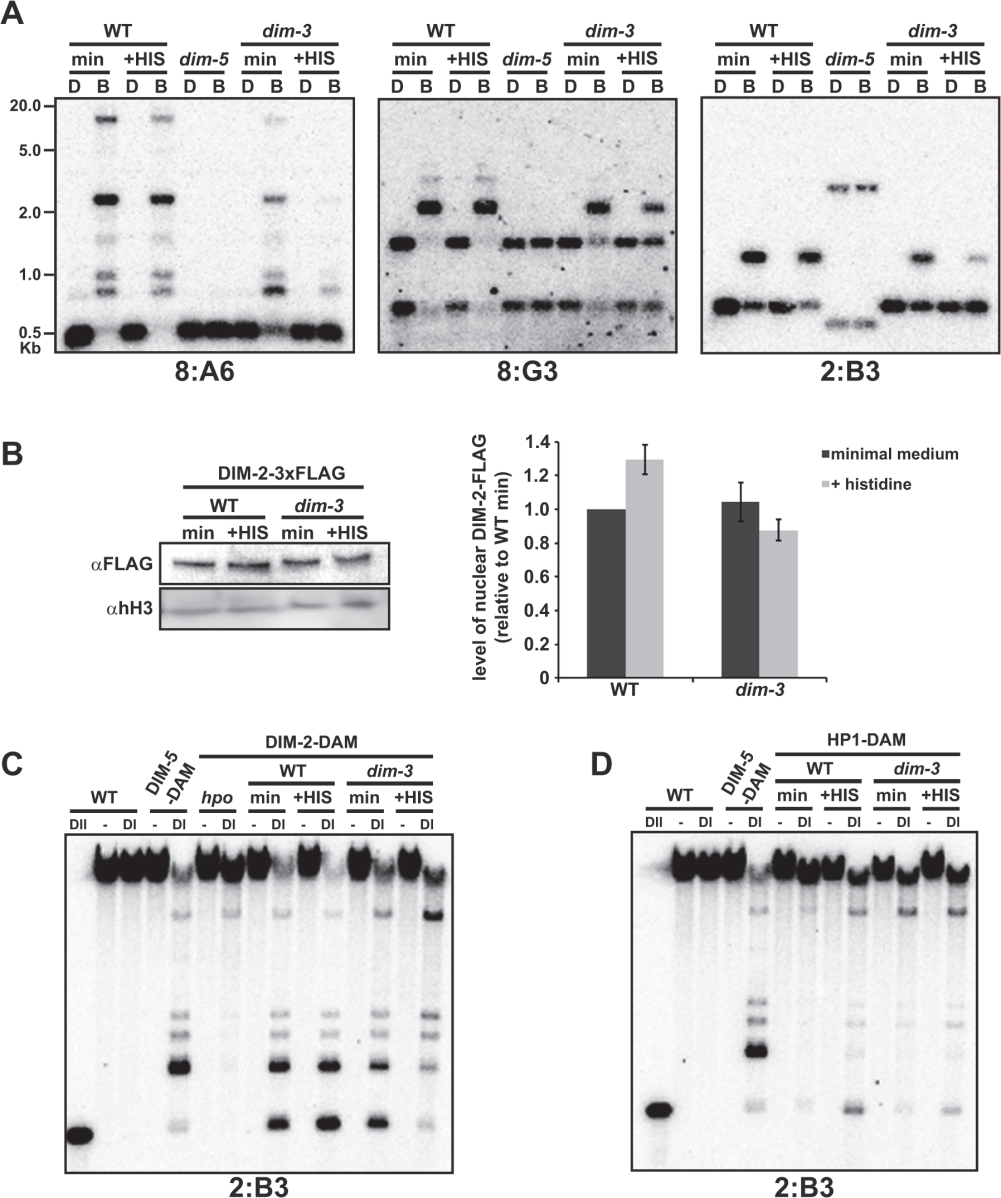
Histidine supplementation exacerbates loss of DNA methylation in *dim-3* strains by displacing DIM-2 from heterochromatin. [A] Southern blot assay (as in Fig. 1A) of DNA methylation in *dim-3* ^+^ (WT) or *dim-3* prototrophic strains grown in minimal or histidine supplemented medium. [B] (left) α-FLAG western blots showing DIM-2-3xFLAG levels in WT and *dim-3* nuclei grown in medium with or without histidine compared with a loading control (histone H3, [hH3]); (right) Quantification of DIM-2-3xFLAG levels from three independent experiments, normalized to hH3 levels. [C-D] Southern blot assay of DamID experiments with WT and *dim-3* strains containing [C] DIM-2-DAM or [D] HP1-DAM, grown with or without histidine, and probed for the heterochromatic region 2:B3. Genomic DNA was digested with DpnI (DI), specifically cutting GA^m^TC sequences or with the A^m^-insensitive isoschizomer DpnII (DII) to monitor complete digestion.

While comparing DNA methylation levels in various *dim-3* strains, we noticed that the histidine-requiring *dim-3* strains had an exacerbated reduction in DNA methylation, leading us to systematically test the possible effect of histidine on DNA methylation in *dim-3* and *dim-3* ^*+*^ strains. We found that histidine supplementation decreased DNA methylation at all heterochromatic regions tested in a *dim-3* strain, as demonstrated by Southern blotting (Fig. 2A) and genome-wide bisulfite sequencing (BS-Seq; Figs. 1B and S1), but has no marked effect on DNA methylation levels in a *dim-3* ^+^ strain.

We tested several possibilities to determine the source of this histidine effect. The "cross pathway control" system causes de-repression of several amino acid biosynthetic pathways concomitant with changes in the level of an individual amino acid [25]. We tested its involvement in the histidine effect by checking for possible consequences of added arginine, lysine, and tryptophan, but found they neither caused loss of DNA methylation nor restored it when added with histidine (S4 Fig.). RNAi components are not required for DNA methylation [11], but histidine supplementation and exposure to other DNA damaging agents, induces expression of the Neurospora Argonaute QDE-2 (EAA31129.2) and increases the levels of qiRNAs (QDE-2-interacting small RNAs; [26]). We tested the possible involvement of DNA damage and RNAi in the histidine effect by growing a *dim-3* strain in medium containing histidine (his), hydroxyurea (HU), or ethyl methane sulfonate (EMS). Only histidine addition reduced DNA methylation in *dim-3* strains (S5A Fig.), and this effect also occurred in a Δ*qde-2* strain (S5B Fig.), indicating the histidine effect in *dim-3* strains involves neither DNA damage nor qiRNAs.

Considering that NUP-6 is critical for nuclear transport and that histidine does not further deplete H3K9me3 levels (below), we investigated whether the histidine effect results from altered nuclear transport and/or sub-nuclear localization of DIM-2 (AF348971.1) or HP1 (AY363166.1), which operate downstream of H3K9me3. We found no significant difference in levels of DIM-2-3xFLAG in nuclei from a *dim-3* strain grown in the presence or absence of histidine relative to WT (Fig. 2B), suggesting that neither histidine nor the *nup-6* ^*dim-3*^ allele impacts DIM-2 nuclear shuttling. In addition, cytological analyses of HP1-GFP (S6 Fig.; [11]) and DamID analyses of HP1-DAM (below) revealed no evidence of a transport defect in *dim-3* strains.

We next tested if histidine perturbs sub-nuclear targeting of DIM-2 or HP1 in a *dim-3* strain. The localization of many heterochromatin-specific proteins, including DIM-2, are not readily detected by standard chromatin immunoprecipitation (ChIP), perhaps due to transient chromatin interactions, but can be detected by DamID [8]. Therefore, we fused the DNA adenine methyltransferase (*dam*) gene to the downstream ends of the *dim-2* [27] and *hpo* (encoding HP1) genes, expressed these constructs in *dim-3* and *dim-3* ^*+*^ strains grown in the presence or absence of supplemented histidine, and tested their localization by digestion of genomic DNA with the GA^m^TC-specific restriction endonuclease DpnI followed by Southern blotting. DIM-2-DAM was found to localize to heterochromatin in both *dim-3* ^+^ and *dim-3* strains grown in minimum medium (Figs. 2C and S7A). In contrast, in a *dim-3* strain grown with histidine, DIM-2-DAM showed reduced localization to heterochromatin, while histidine did not alter DIM-2-DAM localization in a *dim-3* ^*+*^ strain (Figs. 2C and S7A). Unlike the situation with DIM-2, histidine did not reduce, and in fact slightly increased, the heterochromatic localization of HP1-DAM (Figs. 2D and S7B). These findings suggest that in *dim-3* strains, histidine may compromise the direct interaction between DIM-2 and HP1 that is necessary for DNA methylation in Neurospora [10].

### The *dim-3* strain has a global reduction of H3K9me3

**Fig 3:**
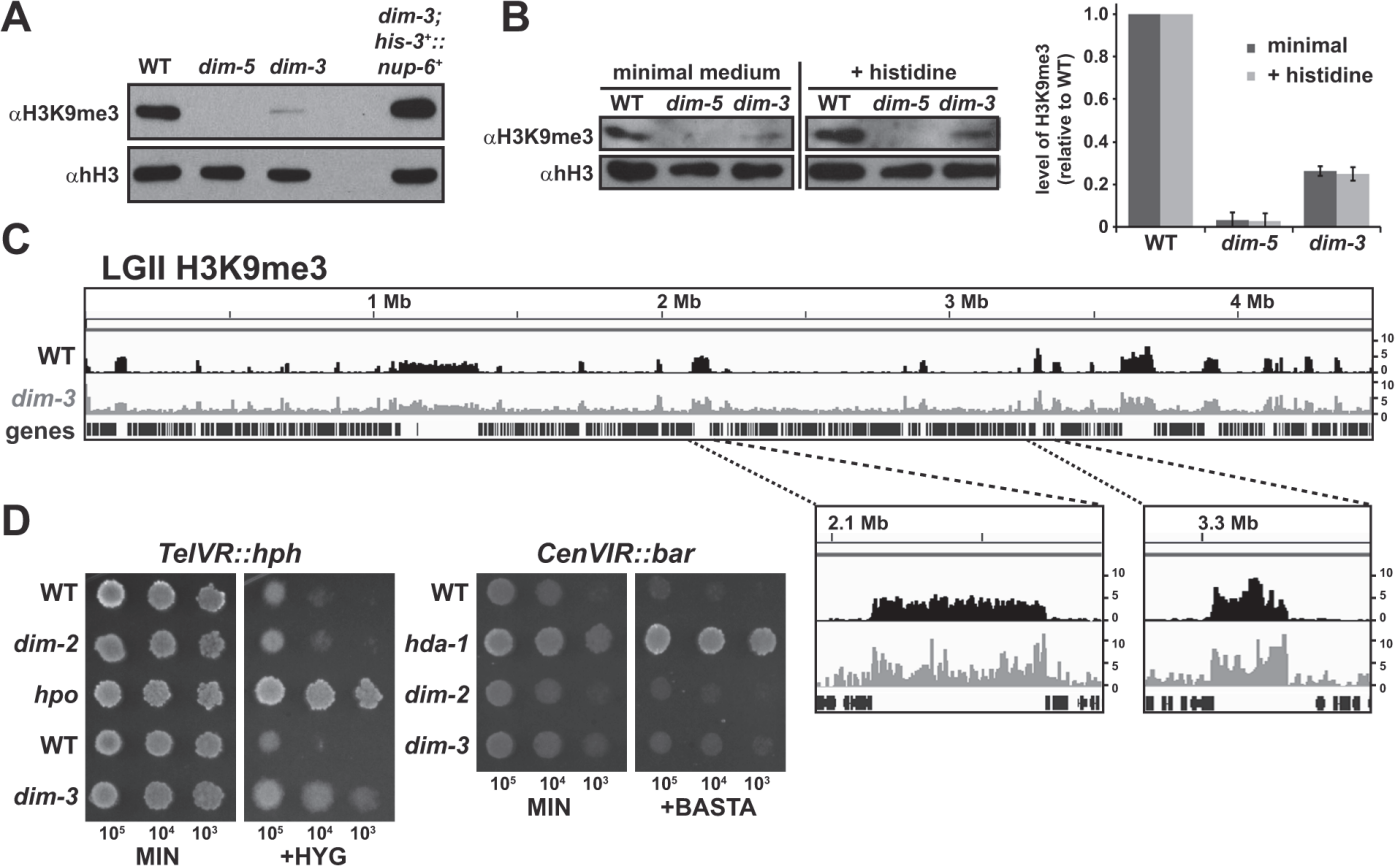
Global reduction of H3K9me3 in *dim-3*compromises telomeric silencing. [A] Western blots of histones isolated from indicated strains were probed for H3K9me3, or histone H3 (hH3) as a loading control. [B] (Left) Western blots of histones isolated from the indicated strains grown with or without histidine probed for H3K9me3 or hH3. (Right) Quantification of H3K9me3 levels from three independent experiments, normalized to hH3 levels. [C] Chromatin Immunoprecipitation sequencing (ChIP-seq) of H3K9me3 from *dim-3* ^+^ (WT) [56] and *dim-3* strains displayed on IGV, as in Fig. 1B. Due to the vastly reduced trimethylation of H3K9 in the *dim-3* strain, this strain shows a reduced signal-to-noise ratio, rendering background more prominent. [D] Growth of strains containing the *telVR*::*hph* or *cenVIR*::*bar* reporter cassettes on minimum medium (MIN) plates or plates supplemented with hygromycin (200 μg/mL, +HYG, left) or phosphinothricin (8 mg/mL, +BASTA, right). Approximate numbers of spotted conidia indicated below the pictures.

To determine whether the reduced DNA methylation observed in *dim-3* strains reflects a loss of H3K9me3, we assessed global H3K9me3 levels by western blotting. H3K9me3 was greatly reduced in *dim-3* strains compared to that in a WT strain, and was reestablished after introduction of an ectopic *nup-6* ^*+*^ gene (Fig. 3A), indicating that NUP-6 is required for normal levels of H3K9me3. Considering that added histidine reduced DNA methylation levels in *dim-3* strains (Fig. 2), we checked if histidine exacerbates the H3K9me3 loss. Western blots showed no additional loss of H3K9me3 (Fig. 3B), supporting the notion that histidine reduces DNA methylation by compromising DIM-2 localization in a *dim-3* background.

To determine if the residual H3K9me3, like DNA methylation, is found in normal heterochromatic regions in *dim-3* strains, we carried out H3K9me3-specific ChIP with high throughput sequencing of associated DNA (ChIP-seq), and despite low signal, found an apparently equivalent distribution of this chromatin mark as in a *dim-3* ^+^ strain, indicating that the remaining H3K9me3 is correctly localized to heterochromatin in a *dim-3* strain (Figs. 3C and S8). Presumably, decreased DNA methylation in *dim-3* strains is due to the dramatic reduction of H3K9me3 in heterochromatin.

### The *dim-3* mutation causes loss of telomeric silencing

To determine if the H3K9me3 loss in *dim-3* strains might compromise heterochromatin-associated silencing, we tested the expression of drug resistance markers integrated at telomeric (*telVR*::*hph*) and centromeric (*cenVIR*::*bar*) sites, which are silent even in the absence of DNA methylation, *i*.*e*. in a *dim-2* mutant [27,28]. The *dim-3* allele de-repressed the *telVR*::*hph* marker as evidenced by growth in the presence of hygromycin (Fig. 3D), indicating that heterochromatin in *dim-3* is compromised, although this de-repression is not as striking as in an *hpo* mutant, which fully de-represses the marker [28]. The *dim-3* allele did not de-repress the *cenVIR*::*bar* marker (Fig. 3D), consistent with the existence of a DNA methylation-independent mechanism for silencing centromeric heterochromatin [27].

### Nuclear transport of the H3K9me3 machinery is not deficient in *dim-3* strains

**Fig 4:**
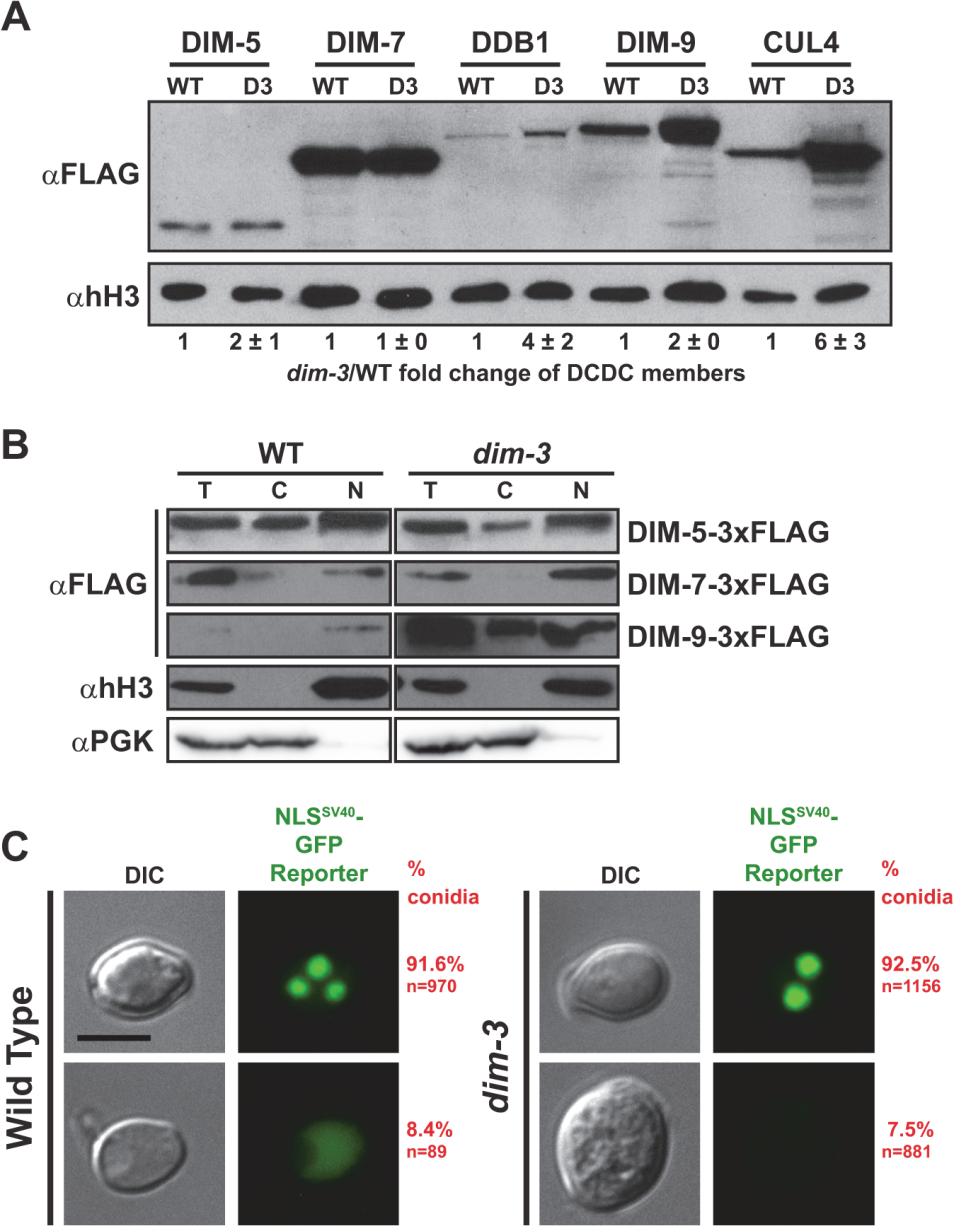
Nuclear transport of DCDC components is unaffected by the *dim-3* mutation. [A] α-FLAG and α-histone H3 (hH3; loading control) western blots of *dim-3* ^+^ (WT) or *dim-3* nuclei expressing individually FLAG-tagged DCDC components. All strains were grown in the presence of histidine. Average and standard deviation of nuclear FLAG-tagged protein levels of three experiments are indicated below the representative images shown. [B] α-FLAG western blots of the total (T) proteins, cytoplasmic (C) fraction, and nuclear (N) fraction of nuclei preparations from *dim-3* ^+^ (WT) and *dim-3* strains expressing DIM-5-3xFLAG, DIM-7-3xFLAG, or DIM-9-3xFLAG. Representative α-histone H3 (hH3) and α-PGK blots from the DIM-5-3xFLAG nuclei are shown as nuclear and cytoplasmic fraction controls, respectively; equivalent results were obtained for all nuclei preparations. [C] Representative differential interference contrast (DIC) and GFP fluorescent images of (left) *dim-3* ^*+*^ or (right) *dim-3* strains expressing an overexpressed NLS^SV40^-GFP reporter construct (pCCG::NLS^SV40^::LexADBD::GFP). While just one representative, multinucleate cell is displayed in the figures here and elsewhere in the paper, we visualized numerous vegetative cells, including both conidia and hyphal cells [62]. Percentages of conidia exhibiting each pattern are listed on the right (P value = 0.43, X^2^-test). Scale bar indicates 5μm.

Considering that the canonical function of NUP-6 is to transport proteins into the nucleus, we tested if a *dim-3* strain has diminished nuclear transport of proteins required for heterochromatin formation and of a protein known to depend on the Importin α/β import system. Our observations that DIM-2 and HP1 localize normally in the nucleus (Figs. 2B and S6) and that *dim-3* strains have only a minor growth defect (S2C Fig.) did not support the idea that *dim-3* strains are defective in nuclear shuttling. Nevertheless, because the *dim-3* mutant shows a striking loss of H3K9me3, we tested whether nuclear transport of DCDC components (DIM-5 [AF419248.1], DIM-7 [AL513463.1], DDB1 [EAA33111.1], DIM-9 [XP_956278.2], and CUL4 [XP_957743.2]) is impaired. To monitor nuclear transport, nuclei were isolated from *dim-3* ^*+*^ and *dim-3* strains bearing DCDC members individually 3xFLAG-tagged at their endogenous loci [8,15] and protein levels were assessed by western blotting (Figs. 4A and S9). Nuclei preparations were shown to be clean of cytoplasmic contamination by probing western blots for phosphoglycerate kinase (α-PGK, EAA33194.1; Fig. 4B).

We found that the nuclear level of every component of DCDC was undiminished by the *dim-3* mutation (Figs. 4A and S9). Indeed, some of the components actually showed *increased* nuclear abundance. DIM-5 and DIM-7 showed equivalent levels in *dim-3* ^*+*^ and *dim-3* strains while the nuclear levels of DDB1, DIM-9 and CUL4 were higher in the *dim-3* strain (Figs. 4A and S9). Moreover, an examination of the distribution of DCDC components between the nuclear and cytoplasmic fractions revealed no increase in the relative amount of the proteins in the cytoplasmic fraction (Fig. 4B). DIM-7, which is an exclusively nuclear protein, showed no change in abundance or localization. Similarly, while the nuclear level of DIM-5 increased slightly in the *dim-3* strain (Fig. 4A), the relative nuclear/cytoplasmic distribution of the protein was equivalent in *dim-3* ^*+*^ and *dim-3* strains. DIM-9, which is predominantly nuclear in *dim-3* ^*+*^ strains, showed an increase in both the cytoplasmic and nuclear fractions of the *dim-3* strain (Fig. 4B). It is interesting that the nuclear levels of DDB1, DIM-9, and CUL4 were elevated in *dim-3* strains. The increase in CUL4 only occurred when this strain is supplemented with histidine (compare Figs. 4B and S9), which is consistent with the induction of a DNA damage response, as previously documented [26]. The basis for increased DDB1 and DIM-9 levels in *dim-3* strains is not clear but may reflect a feedback mechanism to regulate the amount of functional DCDC. Results of qRT-PCR analyses of *dim-7*, *ddb1*, and *dim-9* transcripts in *dim-3* ^+^ and *dim-3* strains did not reveal RNA differences that could account for the differences in protein levels (S10 Fig.), suggesting the effect is at the translational or posttranslational level.

To test if the *dim-3* mutation impacted nuclear transport of a protein with a nuclear localization signal (NLS) known to be bound and transported by Importin α, we overexpressed a GFP reporter construct with an N-terminal SV40 monopartite NLS [22,29–32] in *dim-3* ^+^ and *dim-3* strains and examined nuclear GFP signal by fluorescence microscopy. We found that 91.6% of *dim-3* ^+^ cells had strong GFP signal inside their nuclei, and this result was mirrored in a *dim-3* strain, where 92.5% of cells had nuclear GFP (Fig. 4C). Thus, NUP-6^*dim-3*^ effectively transports canonical nuclear cargo.

### Interactions within the DCDC are not decreased in *dim-3* strains

**Fig 5:**
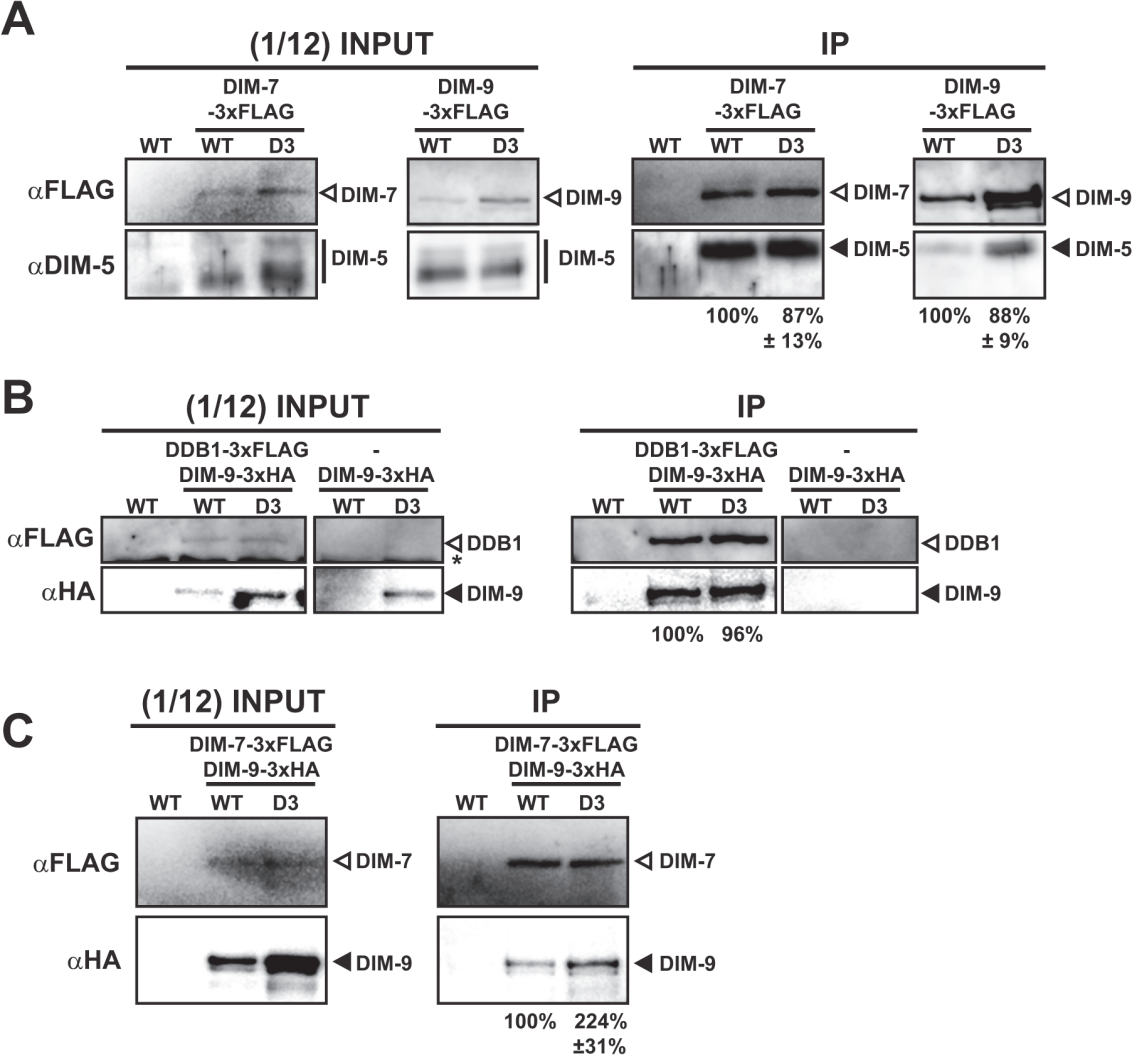
Interactions between DCDC members are not compromised by the mutations in *dim-3*. [A] Western blots detecting DIM-5 interaction with DIM-7-3X-FLAG or DIM-9-3XFLAG from *dim-3* ^+^ (WT) or *dim-3* nuclei. For quantification of purified DIM-5, levels of DIM-7/9-3xFLAG IP from WT and *dim-3* nuclei were equalized and the level of purified DIM-5 from *dim-3* was normalized to the adjusted DIM-7/9-3xFLAG IP level from *dim-3*. The experiment performed in triplicate, and average percent and standard deviation of wild type DIM-5 is noted below. [B] Western blots detecting DIM-9-3xHA interaction with purified DIM-8-3X-FLAG from *dim-3* ^+^ (WT) or *dim-3* nuclei. Experiment performed in duplicate and normalized as in [A], with average noted. Control experiment with DIM-9-3xHA co-IP from WT and *dim-3* nuclei without FLAG-tagged protein demonstrates α-FLAG IP specificity. The star indicates a non-specific band routinely detected in experiments with Neurospora nuclear extracts probed with the rabbit-derived FLAG antibody. [C] Western blots detecting DIM-9-3xHA interaction with purified DIM-7-3X-FLAG from *dim-3* ^+^ (WT) or *dim-3* nuclei. The experiment was performed in triplicate and normalized as in [A].

Given that all DCDC members, as well as DIM-2 and HP1, are transported into the nucleus in *dim-3* strains, we next considered the possibility that NUP-6^*dim-3*^ somehow interferes with DCDC assembly. To test this hypothesis, we monitored DCDC component interactions in *dim-3* and *dim-3* ^*+*^ nuclei by co-immunoprecipitation (co-IP) assays with 3xFLAG-tagged DCDC members. We began by analyzing the interaction between DIM-7 and DIM-5. Equivalent levels of DIM-7-3xFLAG were recovered from *dim-3* and *dim-3* ^*+*^ nuclei (Fig. 5A), consistent with our finding that DIM-7 levels in the nucleus are not affected by the *dim-3* mutation (Figs. 4A and S9). Equivalent levels of DIM-5 were found in co-IPs of both backgrounds, implying that DIM-7 binding to DIM-5 is not compromised in *dim-3* cells (Fig. 5A). Since the DIM-5 interaction with the DCDC members appears mediated through DIM-7 [15], we assessed DIM-5 binding to DIM-9-3xFLAG in *dim-3* ^+^ and *dim-3* nuclei. Because more DIM-9-3xFLAG was recovered from *dim-3* nuclei than from *dim-3* ^+^ nuclei (Fig. 5A), we normalized the amount of DIM-5 purified to the DIM-9-3xFLAG bait levels. The DIM-5 level directly correlated with the DIM-9-3xFLAG level (Fig. 5A), indicating that DIM-5 is not limiting and this interaction is not compromised in *dim-3* cells.

We also monitored DIM-9-3XHA binding to DDB1-3xFLAG. Equal levels of DDB1-3xFLAG were purified from *dim-3* ^+^ and *dim-3* nuclei, and the DIM-9-3xHA interaction appeared normal (Fig. 5B), suggesting no disruption in DCAF-substrate adaptor binding by the *dim-3* mutation. We then monitored the nuclear interaction between DIM-7-3xFLAG and DIM-9-3xHA, which were previously shown to directly interact [15]. DIM-9-3xHA showed an increased interaction with DIM-7-3xFLAG in *dim-3* nuclei (Fig. 5C), potentially because DIM-9 levels are increased in *dim-3* strains (Fig. 5C, input). In summary, all examined DCDC members showed normal or increased interactions in *dim-3* nuclei, implying that the H3K9me3 loss is not due to impaired DCDC assembly in *dim-3* strains.

### DIM-5 and DIM-7 are mislocalized from heterochromatin in *dim-3* strains

**Fig 6:**
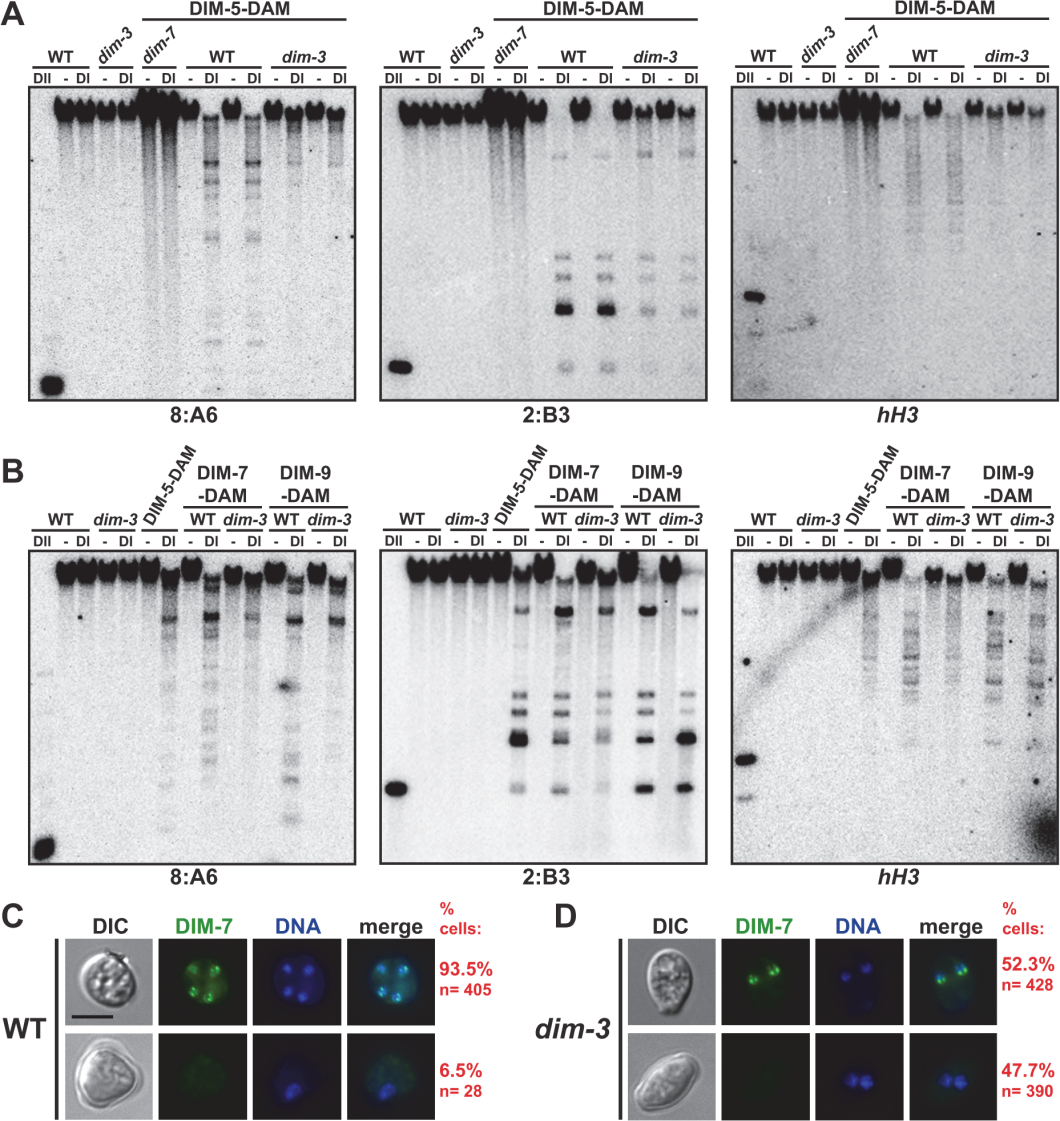
DIM-5 and DIM-7 are mislocalized from heterochromatin in a *dim-3* strain. [A] DamID experiments, as in Fig. 3, with two independent samples of *dim-3* ^+^ (WT) and *dim-3* strains expressing DIM-5-DAM. Representative blots from two heterochromatic regions (8:A6 and 2:B3) and one euchromatic region (*hH3*, encoding histone H3, EAA26767.1) are shown. [B] DamID experiments with DIM-7-DAM and DIM-9-DAM. [C] Representative differential interference contrast (DIC), GFP fluorescent, Hoechst 33342-stained DNA, and merged images of *dim* ^*+*^ strains bearing P_*ccg*_::DIM-7-GFP. Each panel displays one conidium, with four nuclei (top) or two nuclei (bottom) visualized. [D] Representative DIC, GFP, Hoechst 33342-stained DNA, and merged images of *dim-3* strains with P_*ccg*_::DIM-7-GFP; each panel displays one conidium showing two nuclei. Percentages of cells containing at least one sub-nuclear focus are listed on right, and the P-value for loss of focus formation is <1x10^−48^ (X^2^-test). Densely staining DNA foci are thought to represent centromeric heterochromatin [11]. Scale bar indicates 5μm.

It remained possible that the DCDC is not properly localized to heterochromatin in *dim-3* strains. To investigate this possibility, we examined the localization of DIM-5, DIM-7, and DIM-9 in *dim-3* strains by DamID. DIM-5-DAM was previously shown to localize to heterochromatin, and this localization was dependent on DIM-7 [8]. We found that the *dim-3* mutation caused substantially reduced localization of DIM-5-DAM at the three representative heterochromatic regions tested (8:A6, 2:B3, and 8:G3; Figs. 6A and S11A). This reduction was not exacerbated by histidine (S11B Fig.), in contrast to the case for DIM-2 localization (Fig. 2). Probing for euchromatin regions (*hH3* and *pan-1*) revealed that DIM-5-DAM is not misdirected to euchromatin (Figs. 6A and S11A).

The C-terminus of DIM-7 is critical for normal function [8], such that DAM-tagged DIM-7 does not fully complement *dim-7* mutations. Nevertheless, as with DIM-5-DAM, we found that association of DIM-7-DAM with the heterochromatin regions (8:A6, 2:B3, and 8:G3) was also markedly reduced in the *dim-3* background (Figs. 6B and S11C), suggesting that NUP-6^*dim-3*^ impacts targeting of DIM-7-DAM to heterochromatin. In contrast, DamID of DIM-9-DAM did not reveal marked differences between the *dim-3* ^*+*^ and *dim-3* strains (Figs. 6B and S11C). We note that DIM-7-DAM and DIM-9-DAM may show a greater association with the euchromatic gene tested (*hH3*) than did DIM-5-DAM, perhaps reflecting an unknown role of these proteins outside of heterochromatin. The interaction of DIM-7-DAM, but not DIM-9-DAM, with the euchromatin marker was reduced in the *dim-3* strain (Figs. 6A and S11C).

To confirm that DIM-7-DAM is mislocalized, we expressed DIM-7-GFP in *dim-3* ^+^ and *dim-3* vegetative tissue. The level of DIM-7-GFP produced from the native *dim-7* promoter was not cytologically detectable, leading us to express it under the control of the stronger *ccg-1* promoter. Despite overexpression, DIM-7-GFP co-localized with HP1-mCherry (S12 Fig.), at least in the cells examined (conidia), suggesting that DIM-7-GFP behaves normally to mark heterochromatic regions *in vivo* despite the importance of the DIM-7 C-terminus [8]. In 93.5% of *dim-3* ^*+*^ cells DIM-7-GFP formed compact foci, typically at or near the nuclear periphery (Figs. 6C and S12), consistent with DIM-7-GFP marking heterochromatic regions. Interestingly, approximately half of *dim-3* nuclei examined also showed such foci (Fig. 6D). It would be interesting to learn whether the observed difference between *dim-3* and *dim-3* ^*+*^ cells reflect differences in cell cycles of these strains. Unfortunately, because no genetic method to synchronize Neurospora has been developed, our studies were limited to unsynchronized cells. DIM-7-GFP appears equivalently expressed and transported into nuclei of *dim-3* ^*+*^ and *dim-3* strains (S13 Fig.). Thus, the *dim-3* mutation seems to partially perturb normal localization of DIM-7 fusion proteins within the nucleus.

### NUP-6^*dim-3*^ is predominantly delocalized from the nuclear periphery

**Fig 7:**
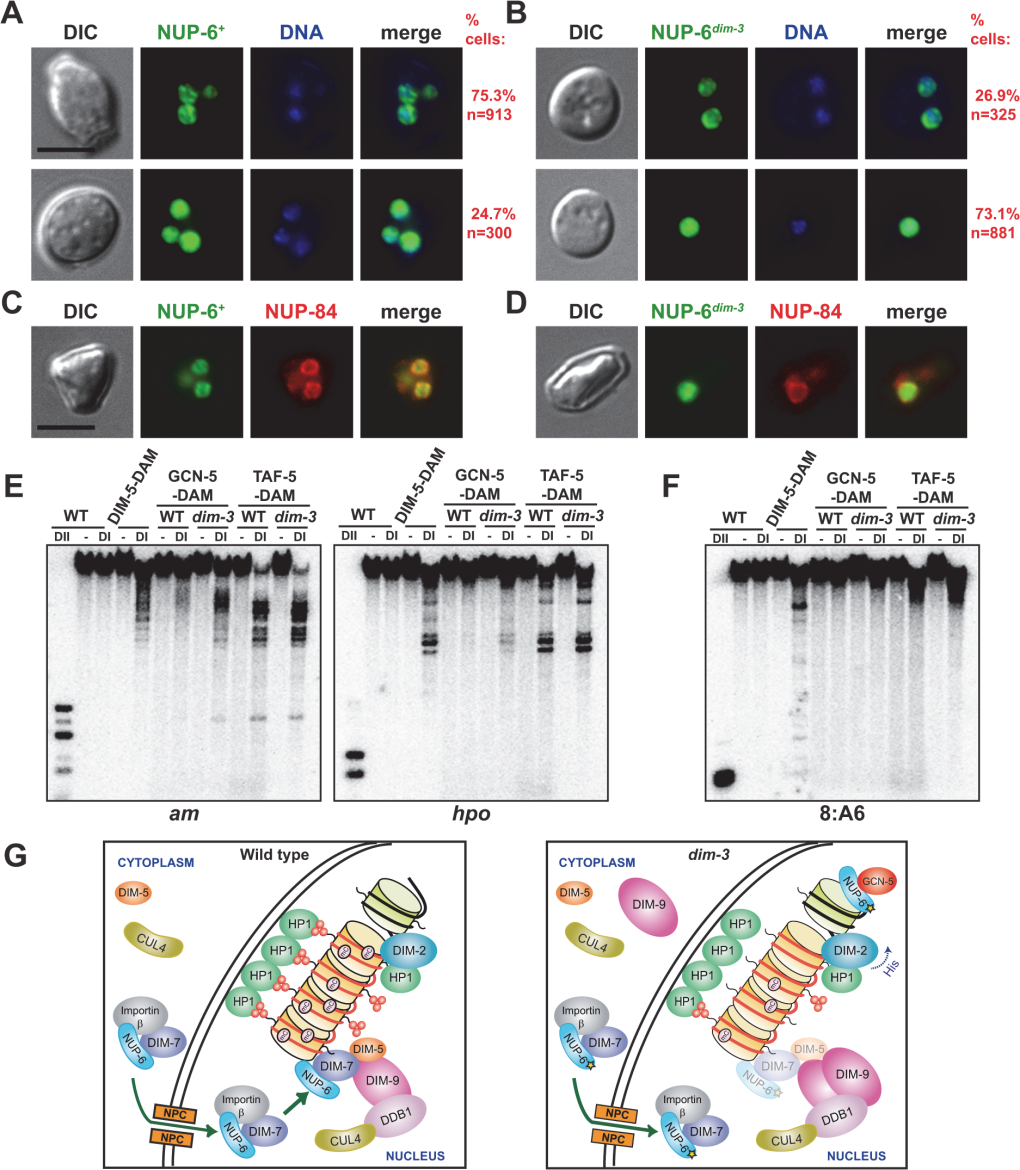
Abnormal localization of NUP-6^*dim-3*^ and increased euchromatic localization of SAGA. [A-B] Representative differential interference contrast (DIC), GFP fluorescent, Hoechst33342 stained DNA, and merged images of [A] *dim-3* ^*+*^ cells expressing NUP-6-GFP or [B] *dim-3* cells expressing NUP-6^*dim-3*^-GFP. Each panel displays one conidium, with one to three nuclei visualized. Top panels show examples of localization at nuclear periphery and bottom panels show examples of dispersed nuclear localization. Percentages of conidia exhibiting each pattern are listed on the right; the P value for loss of membrane localization is <1x10^−124^ (X^2^-test). Densely staining DNA foci are thought to represent centromeric heterochromatin [11]. Scale bar indicates 5 μm. [C-D] Differential interference contrast (DIC), GFP fluorescence, mCherry fluorescence, and merged images of strains expressing the nuclear pore complex member NUP-84-mCherry and [C] NUP-6-GFP or [D] NUP-6^*dim-3*^-GFP. Scale bar indicates 5 μm. For C, one conidium with three nuclei (one out of the focal plane) is displayed. For D, a conidium with one nucleus is displayed. [E-F] DamID experiment, as in Fig. 3, of SAGA complex members GCN-5-DAM and TAF-5-DAM in *dim-3* ^*+*^ (WT) and *dim-3* strains, probed for [E] euchromatic regions (*am*, *hpo*) or [F] a heterochromatic region (8:A6). [G] Illustrations depicting how heterochromatin and DNA methylation depend, at least in part, on a nuclear transport-independent role of NUP-6. In wild type strains (left), DCDC components are hypothetically transported into the nucleus by the NUP-6/Importin β dimer through the Nuclear Pore Complex (NPC), and NUP-6 facilitates formation of active DCDC, which then catalyzes H3K9 methylation. HP1 binds H3K9me3 and directly recruits DIM-2, catalyzing DNA methylation (mC). In the *dim-3* mutant (right), decreased H3K9me3 and DNA methylation at heterochromatin results from diminished localization of DIM-7 and DIM-5 to A-T-rich DNA, despite appropriate nuclear transport. Remaining H3K9me3 is bound by HP1, which efficiently recruits DIM-2 if histidine is absent. *Dim-3* strains also exhibit an increase in nuclear DIM-9 and an increase in GCN-5 targeting to euchromatin.

The cytological mislocalization of DIM-7-GFP in a *dim-3* mutant led us to examine the distribution of NUP-6 and NUP-6^*dim-3*^ as well. Our expectation, based on work in other systems [31,33,34], was that NUP-6 would be largely associated with the nuclear periphery. Indeed, at least in conidia, the majority of NUP-6-GFP was nuclear, but consistent with its role in nuclear transport, a small percentage was observed in the cytoplasm. Most (75.3%) *dim-3* ^*+*^ cells showed NUP-6-GFP localization near the nuclear membrane (NM), forming either foci or a contiguous ring surrounding the genomic DNA (Fig. 7A, upper panel; additional examples of NUP-6-GFP localized at the nuclear periphery are provided in S14 Fig.; Z-stack fluorescent images in S1 Movie). We note that the vast majority of the NM-associated NUP-6 does not co-localize with HP1-marked heterochromatin (S15 Fig.) although some overlap of fluorescent signals was occasionally observed (yellow arrows) leaving open the possibility that NUP-6 is transiently associated with heterochromatin. A minority of *dim-3* ^*+*^ cells (24.7%) show diffuse NUP-6-GFP in the nucleus (Fig. 7A, lower panel). We do not know the basis for the variability in nuclear localization of NUP-6^+^-GFP but it is noteworthy that, because of technical limitations, our study used unsynchronized cells, leaving open the possibility that the protein is differentially distributed in the cell cycle and that the *dim-3* mutation affects the cell cycle.

In contrast to the case in *dim-3* ^*+*^ cells, a majority of *dim-3* conidia (73.1%) showed diffuse localization of NUP-6^*dim-3*^-GFP in the nucleus (Fig. 7B, lower panel; additional examples of dispersed, nuclear NUP-6^*dim-3*^-GFP are provided in S16 Fig.; also evident in Z-stacks of NUP-6^*dim-3*^-GFP, S2 Movie). Nevertheless, 26.9% of *dim-3* conidia still showed compact structures at the nuclear periphery, similar to those observed with wild type NUP-6, consistent with the partial phenotype of *dim-3* strains. The NUP-6^+^-GFP foci were found at the nuclear envelope, as shown by co-localization with a tagged component of the nuclear pore complex, NUP-84-mCherry ([35,36]; XP_964074.1; Fig. 7C); NUP-6^*dim-3*^-GFP was not seen to localize at the nuclear envelope (Fig. 7D). Thus, both NUP-6 and DIM-7 appear to be partially mislocalized from the nuclear envelope in the *dim-3* background, raising the possibility that mislocalization of NUP-6 leads to mislocalization of DIM-7 and other members of DCDC.

### GCN-5 has increased euchromatic localization in a *dim-3* strain

The results presented above suggest that NUP-6 plays a role in the normal localization of DCDC at heterochromatin. To explore the possibility that NUP-6 is also involved in localizing proteins associated with euchromatin, we examined the histone acetyltransferase complex SAGA, which is normally associated with a subset of gene promoters [37,38]. We built C-terminal DAM fusions for two components of the complex, GCN-5 (EDO65389.2), the acetyltransferase subunit, and TAF-5 (XP_961292.2), a structural subunit, and tested their localization by DamID at the euchromatic *am* (amination deficient) gene (EAA32325.1) and the *hpo* gene. Relative to the situation in the wildtype strain, the *dim-3* strain, GCN-5-DAM showed increased association to *am* and *hpo*, while TAF-5-DAM showed no marked change in its association with these genes (Fig. 7E). As expected, these SAGA members were not found at heterochromatin (Fig. 7F). We conclude that NUP-6 differentially influences the localization of members of DCDC and SAGA, representatives of chromatin complexes normally found in heterochromatin and euchromatin, respectively.

## Discussion

Our characterization of the Neurospora *dim-3* mutation, which causes a partial loss of DNA methylation [7], led us to discover an unexpected function for the nuclear transport protein NUP-6 (Importin α). We mapped *dim-3* to a region of Linkage Group V and determined that a mutation causing a glutamic acid to lysine substitution (E396K) in *nup-6* is responsible for the methylation defect. NUP-6 is the Neurospora homolog of the Xenopus / Drosophila Importin α, yeast Srp1p, and human Karyopherin α. As first demonstrated with Xenopus Importin α [39], this protein serves as an adaptor for classical nucleocytoplasmic transport, shuttling proteins from the cytoplasm to the nucleus through the nuclear pore complex [17–19]. We found that in addition to its defect in DNA methylation (Fig. 1A-B), *dim-3* strains have an even more pronounced deficiency in H3K9me3 (Fig. 3A-B) and show reactivation of the silenced telomeric *telVR*::*hph* marker (Fig. 3D), indicating that heterochromatin is compromised in *dim-3* strains. Interestingly, results of DamID suggest that both HP1 and DIM-2 can appropriately localize to heterochromatin in *dim-3* strains grown in minimal medium (Fig. 2C-D). Apparently the reduced H3K9me3 levels in *dim-3* strains still allow nucleation of near-wild type levels of HP1, which in turn recruits DIM-2, resulting in only moderately reduced DNA methylation (Fig. 2A). Histidine supplementation exacerbates loss of DNA methylation in the *dim-3* mutant (Fig. 3A) and results in mislocalization of DIM-2 (Fig. 2C) without causing further reduction of H3K9me3 or loss of HP1 association (Figs. 3B, 2D and S7B). This curious histidine effect, which is not found with wild type strains and is independent of induction of DNA repair processes by histidine [26] and other agents, likely results by compromising the DIM-2—HP1 interaction or by perturbing DIM-2 activity.

Considering that H3K9me3 depends on a five protein complex, DCDC (DIM-5/-7/-9, CUL4, DDB1 Complex), it seemed likely that the reduced tri-methylation of H3K9 caused by the *dim-3* mutation resulted from defective nuclear transport of at least one member of the DCDC. Surprisingly, we observed normal, or increased, nuclear levels of all DCDC components (Figs. 4A-B and S9). Similarly, HP1 and DIM-2, which are downstream of H3K9me3 in the DNA methylation pathway, showed no reduction in nuclei (Figs. 2B and S6). Likewise, a canonical NLS-containing import substrate was effectively transported into *dim-3* nuclei (Fig. 4C). Together, these observations suggest that NUP-6^*dim-3*^ has no nucleocytoplasmic transport defect.

Because NUP-6^*dim-3*^ does not appear to compromise nucleocytoplasmic transport of the DNA methylation machinery, we investigated whether NUP-6 is involved in targeting DCDC to heterochromatin. DamID experiments revealed that DIM-7 and DIM-5 association with heterochromatin is reduced in a *dim-3* strain (Fig. 6A-B). Moreover, cytological examination of DIM-7-GFP showed a marked reduction in foci in the *dim-3* background (Fig. 6C-D) despite being equivalently expressed and transported in the different backgrounds (S13 Fig.), suggesting that NUP-6 is necessary for proper localization of one or more members of the DCDC. This mislocalization presumably results in other defects noted in *dim-3* strains, including the large reduction in H3K9me3 (Fig. 3A-C), the reduced HP1-GFP localization (S6 Fig.), and the loss of *telVR*::*hph* silencing (Fig. 3D). Interestingly, despite being mislocalized, the DIM-5 and DIM-7 interactions with other DCDC components appear normal (Fig. 5), suggesting that formation of this complex does not depend on localization at its normal sub-nuclear site. Since the remaining H3K9me3 in *dim-3* is only found at its normal locations (Fig. 3C), it is conceivable that the mislocalized DCDC does not tri-methylate H3K9 because the DCDC requires some signal from the sequences that underlie heterochromatin for its activity. The observation that DIM-9 localization to heterochromatin was only minimally disrupted in a *dim-3* strain (Fig. 6B), raises the possibility that DIM-9, DDB1, and CUL4 use a different mechanism from that of DIM-5 and DIM-7 for heterochromatin targeting to form the DCDC.

We conclude that *dim-3* strains are not defective in nuclear transport, but are partially defective in localizing some of the heterochromatin machinery. We were curious whether euchromatin machinery might also be affected, either positively or negatively. DamID analyses of two members of the SAGA histone acetyltransferase complex revealed increased localization of one of the proteins (GCN-5) to euchromatin loci in the *dim-3* background (Fig. 7C). Interestingly, studies in Drosophila demonstrated an interaction between Importin α and the chromatin insulator protein CTCF [40].

There have been previous clues from *S*. *cerevisiae* and Xenopus of nuclear transport-independent roles of Importin α homologs. In yeast, *srp1* was originally isolated as a dominant suppressor of ribosomal RNA-specific Polymerase I mutations [33], yet two of the characterized *srp1* alleles had markedly different phenotypes that showed intragenic complementation [41,42]. The *srp1-31* allele is defective in the nuclear transport of mitotic control proteins [43,44], whereas the *srp1-49* allele is proficient for nuclear transport, but shows defects in nucleolus formation and proteasome function [41,42]. In Xenopus, Importin α inhibits the onset of mitosis by binding the mitotic-promotion factor TPX2 in non-mitotic cell cycle stages [32].

Nuclear transport-independent functions of NUP-6 may require a stable localization to specific sub-nuclear regions. In Xenopus, nuclear membrane (NM) associated Importin α promotes nuclear envelope formation independent of the nuclear transport components Importin β, Ran, or CAS [45], and in yeast, Srp1p is in proximity of the NM [31,33,34]. We observed a similar localization for NUP-6^+^-GFP in Neurospora conidia (Fig. 7A), while NUP-6^*dim-3*^-GFP was predominantly found away from the NM (Fig. 7B); the localization of NUP-6-GFP at the NM may vary with stages of the cell cycle and might be differentially controlled in individual cells, possibilities we cannot currently address. The apparent association of NUP-6 with the NM might be mediated through an interaction with the nuclear pore complex (NPC), as we found that NUP-6^+^-GFP co-localized with the NPC member NUP-84 (Fig. 7C). An association of NUP-6 with the inner NM could provide a platform to target chromatin complexes (Fig. 7G, left). We speculate that the *dim-3* change in NUP-6 compromises this targeting (Fig. 7G, right). Of possible relevance to our findings suggesting a nuclear transport-independent function of NUP-6 / Importin α for heterochromatin formation and DNA methylation, the yeast NPC member Nup170p was recently reported to directly interact with chromatin, including the nucleolar-ribosomal DNA repeats and sub-telomeric regions to repress transcription [46]. Other nucleocytoplasmic-independent roles for the nuclear trafficking machinery have been suggested, including targeting Argonaut proteins to cytoplasmic P bodies for miRNA-mediated gene silencing by the human Importin β homolog, Importin-8 [47]. It is also noteworthy that NPC members, including Nup98, Sec13, and Nup50, have been found in NPC-independent nucleoplasm “pools” that directly interact with promoters of developmental or cell cycle genes to activate transcription and increase epigenetic memory through a transport-independent mechanism [48–50]. It will interesting to learn if NUP-6, nucleoporins, or other components of nucleocytoplasmic transport machinery or the NPC mediate heterochromatin formation, DNA methylation, and other functions through transport-independent processes in higher eukaryotes.

## Materials and Methods

Detailed protocols (S1 Text), list of strains (S1 Table), and oligonucleotide sequences (S2 Table) are available online.

### Neurospora growth, strain building, and plasmid construction

*N*. *crassa* strains were grown, maintained, and crossed following standard protocols [51]. All nucleic acid manipulation protocols are given in S1 Text. Gene replacement constructs were generated as described [52] with modifications detailed in S1 Text.

### Nucleic acid and protein manipulations

Genomic DNA isolation (modified from [53]) and Southern blotting [54] were performed as previously described. Primers to amplify DNA from methylated or control regions are listed in S2 Table or [20]. Whole genome sequencing was performed as previously described [55].

ChIP-seq was performed as previously described [56]. To display ChIP-seq data, sequencing reads were mapped to the Neurospora genome using Bowtie 2 [57]. Bam files were converted to read density tdf files spanning 200bp windows using the count function of Integrative Genomics Viewer’s (IGV) IGVtools, and were displayed using the IGV (www.broadinstitute.org/igv/; [58]). H3K9me3 ChIP-seq from a wild type strain has been previously described [56]; for the H3K9me3 ChIP-seq from *dim-3*, 11,662,061 reads (77.63% of total) mapped at least once to the Neurospora genome. The Y-axis shows the number of mapped reads at each position multiplied by 10^6^ and divided by the total number of mapped reads [58], providing normalization between samples.

For bisulfite sequencing, genomic DNA was isolated from strains grown at 32°C with shaking for ∼48 hours in Vogel’s liquid medium, and 50ng was treated with bisulfite using the EZ DNA Methylation Lightning kit (ZYMO Research). Libraries were prepared for sequencing with the EpiGnome Methyl-Seq Kit and EpiGnome Index PCR Primers (Epicentre), purified with Agencourt AMPure XP beads (Beckman Coulter), Qubit HS Assay quantified (Life Technologies), visualized on the Fragment Analyzer (Advanced Analytical), and sequenced on a Illumina HiSeq 2000 (www.illumina.com) at the UO Genomics Core Facility as single-end 100nt reads. The BRAT-BW software package (compbio.cs.ucr.edu/brat/; [59]) was used to prepare and map the reads to the *N*. *crassa* OR74A (annotation NC12) genome, which was converted to a four stranded reference genome to permit bisulfite mapping. BRAT-BW acgt-count “-B” option cytosine-only files produced for the forward and reverse strand reads were merged. A python script (bidensity) was written to calculate the average 5^m^C level over a specified sliding window across the genome, producing a .wig file for IGV display.

For qRT-PCR analyses, RNA was isolated as previously described [60], Qubit-RNA assay quantified (Life Technologies), and equal levels of RNA were DNAse treated (Invitrogen) and the first strand cDNA was synthesized with Superscript III Kit (Life Technologies) following the manufacturers’ protocol. Diluted cDNA was used for triplicate quantitative real-time PCR experiments using FAST SYBR Green master mix (KAPA) with the primers 4701/4702 (*dim-7*), 4661/4662 (*ddb-1*), and 4663/4664 (*dim-9*), and normalized to *actin* (Accession number U78026.1; primers 3209, 3210) (S2 Table) on a Step One Plus Real Time PCR System (Life Technologies).

Immunoprecipitation and western blotting was performed as previously described [52] using antibodies listed in S1 Text. Extraction of histones was performed as previously described [61].

### Accession numbers

Data files of H3K9me3 ChIP-seq and Bisulfite-seq have been deposited to GEO (http://www.ncbi.nlm.nih.gov/geo/) under the accession numbers (GSE61173; ChIP-seq) and (GSE61174; BS-seq). The accession number GSE61175 reports both data sets.

## Supporting Information

S1 Fig *dim-3* has a genome wide loss of DNA methylation which is exacerbated by histidine. Bisulfite sequencing (BS-seq) demonstrating the genome-wide cytosine methylation in *dim-3* ^+^ (wildtype; “WT”, black track) and *dim-3* strains (grown in minimal medium = red track; grown in medium containing histidine = blue track) displayed by Integrative Genomics Viewer, with y-axis denoting the number of normalized mapped reads (reads*1x10^6^/total number of mapped reads; [58]. Genes are displayed on the x-axis below the DNA methylation peaks as green vertical lines, while distances (in Megabases) from the left end of each Linkage Group (LG) are displayed above the graph. Due to the reduced cytosine methylation in the *dim-3* samples, the signal-to-noise ratio is lower, which renders the background more prominent.
(EPS)

S2 Fig NUP-6 structure; NUP-6^*dim-3*^ has normal expression and nuclear transport; *dim-3* strains exhibit slower growth rates. [A] Structural model of NUP-6 (Importin α; [22] shown in “ribbons” graphic, with N- and C-termini labeled. Residues found to be mutated in the *dim-3* strain are colored red (E396K, the causative mutation) and orange (R469H) and displayed as “ball and stick” graphics on the structure. Modeling was done with Pymol (www.pymol.org). [B] Total (T) extract, as well as the cytoplasmic (C) and nuclear (N) fractions from strains expressing NUP-6^+^-3xFLAG or NUP-6^*dim-3*^–3xFLAG, as well as a strain lacking any 3xFLAG-tagged protein, were analyzed by standard western procedures, blotting for the FLAG tagged protein as well as control cytoplasmic (phosphoglycerate kinase; α-PGK) and nuclear (histone H3) proteins. Quantification of three separate experiments, with error shown, presented as the percent of cytoplasmic and nuclear NUP-6–3xFLAG are graphed below. [C] Isolated nuclei from strains expressing NUP-6^+^-3xFLAG and NUP-6^*dim-3*^–3xFLAG, as well as a strain lacking any 3xFLAG-tagged protein, were analyzed by standard western procedures, blotting for the FLAG tagged protein or the histone H3 loading control. The reproducible higher mobility of NUP-6^*dim-3*^–3xFLAG might be indicative of post-translational modification(s), as Xenopus Importin α has been reported to have multiple phosphorylation sites [45]. Quantification of three separate experiments, with error shown, presented as the ratio of NUP-6 protein normalized to the nuclear level of NUP-6^+^-3xFLAG are shown below. [D] Linear growth rate (centimeters per hour; cm/hr) of four wild type Neurospora strains (N5622-N5625, black; average growth rate = 0.36 ±0.004 cm/hr, average R^2^ = 0.99) and four *dim-3* strains (N5628-N5631, red; average growth rate = 0.20 ±0.01 cm/hr, average R^2^ = 0.99), as measured by race tube growth.
(EPS)

S3 Fig Reintroduction of the *dim-3* allele or the E396K mutation cause a loss of DNA methylation. Heterochromatic (8:G3 or 2:B3) or euchromatic (*am*) region Southern blot assays (as in Fig. 1A) of indicated mutations reintroduced into a *dim-3* ^+^ (WT) strain, along with methylation mutant (*dim-5*, *dim-3*) and WT controls. “*dim-3*” strain has both mutations reintroduced while “WT” control shows the effect of just the hygromycin targeting cassette.
(EPS)

S4 Fig Alanine and methionine, but not other amino acids, negate the histidine effect on DNA methylation. Southern blot of genomic DNA digested with the 5^m^C-sensitive enzyme AvaII to assess DNA methylation levels in a *dim-3* ^+^ (WT) strain supplemented with histidine, or *dim-3* strains supplemented with additional amino acids, as listed, or those same amino acids in tandem with histidine. Supplemented methionine presumably restores DNA methylation by increasing internal S-adenosylmethionine (SAM) levels, as shown previously [63]. Southern blotting is done as Fig. 1A.
(EPS)

S5 Fig Histidine specifically causes a loss of DNA methylation in *dim-3*. (A) Southern blot of genomic DNA digested with the 5^m^C-sensitive enzyme AvaII to assess DNA methylation levels in *dim-3* ^+^ (WT) or *dim-3* prototrophic strains grown in the absence of chemicals (-) or in the presence of histidine (HIS), hydroxyuridine (HU) or ethylmethylsulfanate (EMS). Note that all of these chemicals induce QDE-2 interacting RNAs (qiRNAs), which reportedly are indicative DNA damage or a compromised genome [26]. Southern blotting is done as Fig. 1A. (B) Southern blot of genomic DNA digested with the 5^m^C-sensitive enzyme AvaII to assess DNA methylation levels in WT or *dim-3* strains deleted of the Neurospora Argonaut gene, *qde-2*. Southern blotting is done as Fig. 1A.
(EPS)

S6 Fig HP1-GFP is effectively transported into the nucleus in *dim-3*. Differential Interference Contrast (DIC) and fluorescent microscopy images of HP1-GFP expressed in *dim-3* ^+^ (WT) and *dim-3* conidia (one pair shown for each) demonstrating that HP1 is transported into nuclei. Scale bar indicates 5μm. HP1-GFP concentrates as green foci in WT cells (top), which co-localizes with DNA [11], but appears mislocalized (single focus) in *dim-3* nuclei (bottom) as observed with Δ*dim-7* [8], presumably due to reduced H3K9me3 levels [8,11,15,61,64].
(EPS)

S7 Fig Histidine displaces DIM-2-DAM, but not HP1-DAM from heterochromatin. Southern blot assay of DamID experiments with *dim-3* ^+^ (WT) and *dim-3* strains containing [A] DIM-2-DAM or [B] HP1-DAM grown with or without histidine and probed for the heterochromatic region 8:A6 or the euchromatic region *pan-1*. Genomic DNA was digested with DpnI (DI), specifically cutting GA^m^TC sequences or with the A^m^- insensitive isoschizomer DpnII (DII) to monitor complete digestion.
(EPS)

S8 Fig *dim-3* has a genome-wide loss of H3K9me3. Chromatin Immunoprecipitation sequencing (ChIP-seq) of H3K9me3 from *dim-3* ^+^ (wildtype, “WT”, black track) [56] and a *dim-3* strain (grown in minimal medium, light gray track) displayed by Integrative Genomics Viewer, with y-axis denoting the approximate number of normalized mapped reads (reads*1x10^6^/total number of mapped reads; [58]. Genes are displayed on the x-axis below the H3K9me3 peaks as vertical lines, while distances (in Megabases) from the left end of each Linkage Group (LG) are displayed above the graph. Due to the vastly reduced trimethylation of H3K9 in the *dim-3* strain, the signal-to-noise ratio is reduced, rendering the background more prominent.
(EPS)

S9 Fig Nuclear transport of DCDC components is unaffected by the *dim-3* mutation. α-FLAG and α-histone H3 (hH3; loading control) western blots of *dim-3* ^+^ (WT) or *dim-3* nuclei expressing individually FLAG-tagged DCDC components, as in Fig. 4A, except non-auxotrophic strains were grown in minimum medium. Average and standard deviation of nuclear FLAG-tagged protein levels of three experiments are indicated below. Representative images are shown.
(EPS)

S10 Fig Transcription of *dim-7*, *ddb-1*, and *dim-9* is minimally altered in a *dim-3* strain. Fold change of mRNA levels in *dim-3* ^+^ (WT) and *dim-3* strains, as assessed by quantitative RT-PCR using the primers oAK262/ oAK263 (*dim-7*), oAK222/223 (*ddb-1*), and oAK224/225 (*dim-9*), with signal normalized to actin (primers 3209, 3210), is displayed as a bar chart. Standard deviation of three biological replicates, each with at least three technical replicates, is shown. Inset shows a representative ethidium bromide-stained agarose gel image showing comparable levels of input RNA used for the qPCR reaction (doublet of ribosomal RNA shown).
(EPS)

S11 Fig DIM-5-DAM and DIM-7-DAM are mislocalized from heterochromatin, and histidine does not further displace DIM-5-DAM. [A] DamID Southern blot experiments of the heterochromatic region 8:G3, as in Fig. 3 and 6, with two independent samples of *dim-3* ^+^ (WT) and *dim-3* strains expressing DIM-5-DAM. [B] DamID experiments with *dim-3* ^+^ (WT) and *dim-3* expressing DIM-5-DAM grown in minimum medium (min) or medium containing histidine (+HIS). Δ*dim-7*; DIM-5-DAM sample indicates a complete loss of DIM-5-DAM targeting [8]. Southern blots from three heterochromatic regions (8:A6, 2:B3, and 8:G3) and one euchromatic region (*pan-1*) are shown. [C] DamID experiments with DIM-7-DAM and DIM-9-DAM.
(EPS)

S12 Fig DIM-7-GFP co-localizes with HP1-mCherry marked heterochromatin and is near the inner nuclear periphery. (A) Representative differential interference contrast (DIC), GFP fluorescent, mCherry fluorescent, and merged images of P_ccg_::DIM-7-GFP and HP1-mCherry in a WT strain. One conidium with two nuclei is displayed. Scale bar indicates 5μm. (B) Representative differential interference contrast (DIC), GFP fluorescent, mCherry fluorescent, and merged images of P_ccg_::NCA-1-GFP (CAB65295.1), which marks the nuclear and cell membranes [65] and P_ccg_::DIM-7-mCherry in a WT background. One conidium with one nucleus is displayed.
(EPS)

S13 Fig P_ccg_::DIM-7-GFP exhibits equal expression and nuclear transport between *dim-3* ^+^ and *dim-3* strains. [A] α-GFP western blots of whole cell extract of either a *dim-3* ^+^ (WT) strain, or *dim-3* ^+^ or *dim-3* strains expressing Pccg::DIM-7-GFP. Top blot shows the levels of DIM-7-GFP fusion protein (predicted molecular weight: 102.5KDa), while the bottom blot shows the position of free GFP (predicted molecular weight: 26.0KDa). PonceauS-stained membrane serves as a loading control. [B] α-GFP western blot showing the total culture, cytoplasmic fraction, and nuclear fraction levels of overexpressed DIM-7-GFP found in nuclei isolated from *dim-3* ^+^ (WT) or *dim-3* strains expressing this construct. PonceauS-stained membrane of proteins present at the dye front serves as a loading control.
(EPS)

S14 Fig Punctate localization NUP-6-GFP. [A-B] Additional examples, relative to Fig. 7A, of differential interference contrast (DIC), GFP fluorescent, Hoechst33342 stained DNA, and merged images of [A] *dim-3* ^*+*^ cells expressing NUP-6-GFP from the native promoter, or [B] overexpressed NUP-6-GFP using the *ccg* promoter in *dim-3* cells. Each panel displays one conidium, with one to three nuclei. Densely staining DNA foci are thought to represent centromeric heterochromatin [11]. Scale bar indicates 5μm. For overexpressed NUP-6-GFP, 83.6% of conidia (n = 544) exhibited a nuclear periphery localization while 16.4% of conidia (n = 107) had a nuclear localization; the P value for loss of membrane localization is <1x10^–33^ (X^2^-test).
(EPS)

S15 Fig NUP-6-GFP minimally co-localizes with HP1-mCherry-marked heterochromatin. Differential interference contrast (DIC), GFP fluorescent, mCherry fluorescent, and merged images of NUP-6-GFP and HP1-mCherry in a WT strain background. Yellow arrows mark potential co-localization sites. Four independent examples are shown, and scale bar indicates 5μm. For each panel, one conidium showing one or two nuclei is displayed.
(EPS)

S16 Fig Dispersed localization of NUP-6^*dim-3*^-GFP. [A-B] Additional examples of differential interference contrast (DIC), GFP fluorescent, Hoechst33342 stained DNA, and merged images of [A] *dim-3* cells expressing NUP-6^*dim-3*^-GFP from the native promoter, as in Fig. 7B, or [B] overexpressed NUP-6^*dim-3*^-GFP using the *ccg* promoter in *dim-3* ^*+*^ cells. Each panel displays one conidium, with one to three nuclei. Densely staining DNA foci are thought to represent centromeric heterochromatin [11]. Scale bar indicates 5μm. For overexpressed NUP-6^*dim-3*^-GFP, 54.8% of conidia (n = 583) exhibited a nuclear periphery localization while 45.2% of conidia (n = 481) had a nuclear localization; the P value for loss of membrane localization is <1x10^–33^ (X^2^-test).
(EPS)

S1 Movie NUP-6^+^-GFP forms INM structures inside the nucleus. Z-stack image movie of NUP-6-GFP constructed with fluorescent images and compiled using ImageJ. One conidium with three nuclei is displayed.
(AVI)

S2 Movie NUP-6^*dim-3*^-GFP is mislocalized from the INM. Z-stack image movie of NUP-6^*dim-3*^-GFP constructed with fluorescent images and compiled using ImageJ. One conidium with two nuclei is displayed.
(AVI)

S1 Table Strains used in this study. (DOCX)

S2 Table Oligonucleotides used in this study. (DOCX)

S1 Text Detailed Materials and Methods. (DOCX)
