# Supplementary material for: Neurospora Importin α Is Required for Normal Heterochromatic Formation and DNA Methylation
Source: PLoS Genet. 2015 Mar 20;11(3):e1005083. doi: 10.1371/journal.pgen.1005083 (PMC4368784; doi:10.1371/journal.pgen.1005083)
Supplement: S1 Table — (DOCX) [file pgen.1005083.s019.docx]

**Table S1: Strains used in this study**

**Number Genotype Reference**

| N150 | *Wild type* Neurospora crassa*, mat A* | [66] |
| --- | --- | --- |
| N2108 | *dim-3; mat A* | this work |
| N2109 | *dim-3; mat a* | this work |
| N2146 | *dim-3; his-3; mat A* | this work |
| N2194 | *dim-3; his-3; mat a* | this work |
| N2264 | *dim-5; pan-1; leu-2; his-3; mat a* | [14] |
| N2833 | *dim-2; arg-10; TelVR::T::hph; mat A* | [28] |
| N3004 | *hpo^RIP2^; TelVR::T::hph; mat A* | [28] |
| N3120 | *TelVR::T::hph; mat a* | [28] |
| N4905 | *CenVIR::bar;* Δ*dim-2::hph; his-3; trp-2; mat a* | [27] |
| N4906 | *CenVIR::bar;* Δ*hda-1::hph; his-3; trp-2; mat A* | [27] |
| N4909 | *CenVIR::bar; his-3; trp-2; mat a* | [27] |
| N4949 | *his-3^+^::P_ccg_::hpo::10xGly::GFP; mat A* | [11] |
| N4950 | *dim-3; his-3^+^::P_ccg_::hpo::10xGly::GFP; mat A* | this work |
| N4955 | *dim-3; his-3^+^::P_nup-6_::nup-6^+^; mat ?* | this work |
| N4956 | *his-3^+^::P_dim-5_::dim-5:10xGly::dam; mat ?* | [8] |
| N4957 | *dim-3; his-3^+^::P_dim-5_::dim-5::10xGly::dam; mat ?* | this work |
| N4958 | *his-3^+^::P_ccg_::nup-6^+^::10xGly::gfp; dim-3; mat a* | this work |
| N4962 | *his-3^+^::P_ccg_::nup-6^+^::10xGly::gfp; mat a* | this work |
| N4963 | *his-3^+^::P_nup-6_::nup-6^+^::10xGly::3xFLAG; mat a* | this work |
| N4964 | *his-3^+^::P_nup-6_::nup-6^dim-3^::10xGly::3xFLAG; mat ?* | this work |
| N4968 | *his-3^+^::P_nup-6_::nup-6^+^::10xGly::gfp; mat a* | this work |
| N4970 | *his-3^+^::P_nup-6_::nup-6^dim-3^::10xGly::gfp; dim-3; mat a* | this work |
| N4989 | *dim-5::3xflag::10xGly::hph; his-3; mat a* | [15] |
| N4990 | *dim-3; dim-5::10xGly::3xflag::hph; his-3; mat A* | this work |
| N4991 | *dim-7::3xflag::10xGly::hph; mat a* | [15] |
| N4992 | *dim-3; dim-7::10xGly::3xflag::hph; mat a* | this work |
| N4993 | *ddb1::3xflag::10xGly::hph; his-3; mat a* | [15] |
| N4994 | *dim-3; ddb1::10xGly::3xflag::hph; his-3; mat A* | this work |
| N4995 | *dim-9::10xGly::3xflag::hph; mat ?* | [15] |
| N4996 | *dim-3; dim-9::10xGly::3xflag::hph; mat a* | this work |
| N4997 | *his-3^+^::P_cul4_::3xflag::10xGly::cul4; mat A* | [15] |
| N4998 | *dim-3; his-3^+^::P_cul4_::3xflag::10xGly::cul4; mat A* | this work |
| N5173 | *dim-9::10xGly::3xha::hph; ddb1::10xGly::3xflag::hph; his-3; mat ?* | this work |
| N5174 | *dim-3; dim-9::10xGly::3xha::hph; ddb1::10xGly::3xflag::hph; his-3; mat ?* | this work |
| N5175 | *dim-9::10xGly::3xha::hph; dim-7::10xGly::3xflag::hph; his-3?; mat ?* | this work |
| N5176 | *dim-3; dim-9::10xGly::3xha::hph; dim-7::3xflag::10xGly::hph; his-3?; mat ?* | this work |
| N5183 | *his-3^+^::P_ccg_::dim-7::10xGly::gfp; mat A* | this work |
| N5184 | *dim-3; his-3^+^::P_ccg_::dim-7::10xGly::gfp; mat ?* | this work |
| N5216 | *dim-9::10xGly::dam::hph; his-3; mat A* | this work |
| N5217 | *dim-3; dim-9::10xGly::dam::hph; sad-1?; his-3; mat ?* | this work |
| N5220 | *dim-9::10xGly::3xHA::hph; his-3, mat A* | this work |
| N5223 | *dim-7::10xGly::dam::hph; his-3; mat A* | this work |
| N5224 | *dim-3; dim-7::10xGly::dam::hph; sad-1?; his-3; mat ?* | this work |
| N5225 | *hpo::10xGly::dam::hph; mat ?* | this work |
| N5226 | *dim-3; hpo::10xGly::dam::hph; mat ?* | this work |
| N5227 | *dim-2::10xGly::dam::hph; mat ?* | this work |
| N5228 | *dim-3; dim-2::10xGly::dam::hph; mat ?* | this work |
| N5385 | *his-3; nup-6(dim-3)::hph; mus-52::bar?; sad-1?; mat ?* | this work |
| N5386 | *his-3; nup-6(R469H)::hph; mus-52::bar?; sad-1?; mat ?* | this work |
| N5387 | *his-3; nup-6(E396K)::hph; mus-52::bar?; sad-1?; mat ?* | this work |
| N5388 | *his-3; nup-6^+^::hph; mus-52::bar?; sad-1?; mat ?* | this work |
| N5389 | *TelVR::T::hph; mat ?* | this work |
| N5390 | *dim-3; TelVR::T::hph; mat ?* | this work |
| N5391 | *dim-2::10xGly::3xFLAG::hph; mat ?* | this work |
| N5392 | *dim-3; dim-2::10xGly::3xFLAG::hph, mat ?* | this work |
| N5393 | *dim-3; dim-9::10xGly::3xHA::hph; his-3; mat ?* | this work |
| N5398 | *dim-3; CenVIR::bar; mat ?* | this work |
| N5431 | *dim-3;* Δ*qde-2::bml;* Δ*sad-1::hph?; his-3?; mat ?* | this work |
| N5432 | *dim-3;* Δ*qde-2::bml;* Δ*sad-1::hph?; his-3?; mat ?* | this work |
| N5433 | Δ*qde-2::bml;* Δ*sad-1::hph?; his-3?; mat ?* | this work |
| N5434 | Δ*qde-2::bml;* Δ*sad-1::hph?; his-3?; mat ?* | this work |
| N5604 | *gcn-5::10xGly::dam::hph; mus52::bar?; mat ?* | this work |
| N5605 | *dim-3; gcn-5::10xGly::dam::hph; mus52::bar?; mat ?* | this work |
| N5606 | *taf-5::10xGly::dam::hph; mus52::bar?; mat ?* | this work |
| N5607 | *dim-3; taf-5::10xGly::dam::hph; mus52::bar?; mat ?* | this work |
| N5619 | *his-3^+^::P_ccg_::dim-7::10xGly::gfp; hpo::10xGly::mCherry::hph; mat ?* | this work |
| N5622 | *Wild type; mat A* | this work |
| N5623 | *Wild type; mat A* | this work |
| N5624 | *Wild type; mat a* | this work |
| N5625 | *Wild type; mat A* | this work |
| N5628 | *dim-3; mat A* | this work |
| N5629 | *dim-3; mat A* | this work |
| N5630 | *dim-3; mat a* | this work |
| N5631 | *dim-3; mat A* | this work |
| N5659 | *his-3^+^::P_nup-6_::nup-6^+^-gfp; hpo::10xGly::mCherry::hph; mat ?* | this work |
| N5740 | *his-3^+^::P_ccg_::nca-1::gfp; trp-2^-^::P_ccg_::dim-7^+^::mCherry::nat-1; mat ?* | this work |
| N5815 | *his-3^+^::P_ccg_::SV40^NLS^::LexA-DBD::gfp; mat ?* | this work |
| N5816 | *his-3^+^::P_ccg_::SV40^NLS^::LexA-DBD::gfp; mat ?* | this work |
| N5817 | *dim-3; his-3^+^::P_ccg_::SV40^NLS^::LexA-DBD::gfp; mat ?* | this work |
| N5818 | *dim-3; his-3^+^::P_ccg_::SV40^NLS^::LexA-DBD::gfp; mat ?* | this work |
| N5819 | *::PtrpC::hph; ::Pccg::nup-84::10xgly::mCherry; his-3^+^::P_nup-6_::nup-6^+^::10xGly::gfp; mat a* | this work |
| N5820 | *::PtrpC::hph; ::Pccg::nup-84::10xGly::mCherry; his-3^+^::P_nup-6_::nup-6^dim-3^::10xGly::gfp; mat a* | this work |

**Additional Reference**

66. Galagan JE, Calvo SE, Borkovich KA, Selker EU, Read ND, et al. (2003) The genome sequence of the filamentous fungus *Neurospora crassa*. Nature 422: 859-868.
