# Supplementary material for: Neurospora Importin α Is Required for Normal Heterochromatic Formation and DNA Methylation
Source: PLoS Genet. 2015 Mar 20;11(3):e1005083. doi: 10.1371/journal.pgen.1005083 (PMC4368784; doi:10.1371/journal.pgen.1005083)
Supplement: S2 Table — (DOCX) [file pgen.1005083.s020.docx]

**Table S2: Oligonucleotides used in this study**

**Oligo# Gene Sequence**

| 1864 | 8:G3 | 5’AAACGCGTTACGGCTCTTGC |
| --- | --- | --- |
| 1869 | 8:G3 | 5’GTCCGGGTAACTTGATGTGG |
| 1877 | 8:A6 | 5’TGGTTGGTCGATTGTGGTGG |
| 1878 | 8:A6 | 5’TTTTGAGGATCCGCCATCCG |
| 2354 | *hph* | 5’CCGCTCGAGCTATTCCTTTGCCCTCGGAC |
| 3181 | *pan-1* | 5’CGATAAGCTTGATATCGAATTCAGGTTGTCCGGCCATCTCAGTCTGATCC |
| 3182 | *pan-1* | 5’TCGCATACGCCAACCCATGC |
| 3209 | *actin* | 5’AATGGGTCGGGTATGTGCAA |
| 3210 | *actin* | 5’CTTCTGGCCCATACCGATCAT |
| 3628 | *dim-9* | 5’accgcggtggcggccgctctagaactagtaacgaagcgtggcatcgagct |
| 3629 | *dim-9* | 5’tgctatacgaagttatggatccgagctcgtttaagagttctatgtaatcag |
| 3630 | *dim-9* | 5’cctccgcctccgcctccgccgcctccgccgaattcatcaatgtcgcttcc |
| 3631 | *dim-9* | 5’gaggtcgacggtatcgataagcttgatatatcaatgagctcactgtcatg |
| 3642 | *dim-7* | 5’accgcggtggcggccgctctagaactagtacaggagcaattatacca |
| 3643 | *dim-7* | 5’tgctatacgaagttatggatccgagctcgggccttcctgcattattg |
| 3644 | *dim-7* | 5’cctccgcctccgcctccgccgcctccgccaatcaacaaccgacgttt |
| 3645 | *dim-7* | 5’gaggtcgacggtatcgataagcttgatatgcaacctcgggtaggccg |
| 3755 | *NLS-LexADB* | 5' AGGCGCGCCTCCAAAAAAGAAGAGAAAGG |
| 3756 | *NLS-LexADB* | 5' GCTCTAGATTAGGGTTCACCGGCAGCCA |
| 4286 | *nup-6* | 5’GATCGAGCGGCCGCCATGGCGGCGGATGAGTATGTGG |
| 4287 | *nup-6* | 5’GATCGAGAATTCATGGCTAGCACGATGGAAGTCCG |
| 4293 | *nup-6* | 5’GATCGATCTAGAATGGCTGACCGATACATTCCCGAGC |
| 4294 | *nup-6* | 5’GATCGATTAATTAACATGTCCATCGACTCGGTGCCGTTAGC |
| 4307 | *nup-6* | 5’CTCTTAGTAACTTCTGCCGTGG |
| 4309 | *nup-6* | 5’TGCTATACGAAGTTATGGATCCGAGCTCGAATGCTGTACAATCCGTCTAG |
| 4310 | *nup-6* | 5’TTCCCACCTCCTGGACGGCCC |
| 4329 | *nup-6* | 5’GATGCCACCCGGGATCACTATTACATGTCCATCGACTCGGTGC |
| 4342 | *nup-6* | 5’gccgacctcaagactcgtaagAAGgcctgctgggctatcagcaacg |
| 4343 | *nup-6* | 5’CGTTGCTGATAGCCCAGCAGGCCTTCTTACGAGTCTTGAGGTCGGC |
| 4346 | *dim-7* | 5’GATCGAGGATCCATGGCCGGACCAGGGCGCCC |
| 4347 | *dim-7* | 5’GATCGATTAATTAAAATCAACAACCGACGTTTGATCG |
| 4383 | *mCherry* | 5’gatcgattaattaagggcggaggcggcggaggcggaggcggaggcgaggataacatggcc |
| 4384 | *mCherry* | 5’gatcgaGAATTCttacttgtacagctcgtccatgC |
| 4435 | *gcn-5* | 5’CAACAATGATGGCGAACGCG |
| 4436 | *gcn-5* | 5’CCTCCGCCTCCGCCTCCGCCGCCTCCGCCAGGCTCGAGGTGTGACCACTCG |
| 4437 | *gcn-5* | 5’TGCTATACGAAGTTATGGATCCGAGCTCGAAGGGTGAAAGTCGTGAGGACC |
| 4438 | *gcn-5* | 5’GGCTGTACTGCATAGTCGGG |
| 4443 | *taf-5* | 5’TCCATTGCCAACCTGGACCG |
| 4444 | *taf-5* | 5’CCTCCGCCTCCGCCTCCGCCGCCTCCGCCACGCTCAGGATCATAGCATCC |
| 4445 | *taf-5* | 5’TGCTATACGAAGTTATGGATCCGAGCTCGGAGGTTCAGGATCAGCGGTCC |
| 4446 | *taf-5* | 5’AGCAGTGGTATGAAGGCCGG |
| 4449 | *gcn-5* | 5’gatcgaggatccATGCCCGCTAATTCCGTCACG |
| 4451 | *taf-5* | 5’gatcgaggatccATGTCCAATCCCCCACCAGC |
| 4661 | *ddb-1* | 5’CTACGTCGCCCCGATTCACC |
| 4662 | *ddb-1* | 5’TGATGGTCCCGTTGATAACC |
| 4663 | *dim-9* | 5’TGAGCACGAGGATGTAGGCG |
| 4664 | *dim-9* | 5’CGGTCTCGACATAATTCGGG |
| 4701 | *dim-7* | 5’CTTCAAGGACCACGAGGAGC |
| 4702 | *dim-7* | 5’TTGATCGGGAGTGGCAGCGG |
| 4860 | *nup-84* | 5’gatcgaactagtatggcacccacagttgtgagc |
| 4861 | *nup-84* | 5’gatcgattaattaattgtggcttgacagaccaaagctcc |
| 4865 | *trp-2* | 5'gatcgagatatcGGTCTGGATCTCGGAGGCG |
| 4866 | *trp-2* | 5’gatcgacatatgTCATCGGCTCCATCGTCGGC |
| 4867 | *trp-2* | 5’gatcgagcggccgcCTGAACAACTGAACTGTAACG |
| 4878 | *trp-2* | 5’gatcgaccgcggCTAAACTGTATAGTGTCCGG |
| 4879 | *trp-2* | 5’gctccttctaCTGAACAACTGAACTGTAACG |
| 4880 | *P_ccg_* | 5’AGTTGTTCAGtagaaggagcagtccatctgcg |
| 4881 | *mCherry* | 5’atatcagttgttacttgtacagctcgtccatgcc |
| 4882 | *nat1* | 5’gtacaagtaacaactgatattgaaggagc |
| 4883 | *nat1* | 5’aaccccatccgccggtacgcg |
| 4884 | *nat1* | 5’tccttcaccaccgacaccgtcttcc |
| 4885 | *nat1* | 5’TTAGCCAGTCgagctctcaggggcagggcatgc |
| 4886 | *trp-2* | 5’ctgagagctcGACTGGCTAAAGCGAACCGGCC |
| 4958 | *LexADBD -GFP* | 5’CGATGCGTCTAGAATGGCGCCTCCAAAAAAGAAGAGA |
| 4959 | *LexADBD -GFP* | 5’CGATGCGTTAATTAAGGGTTCACCGGCAGCCACACG |
| hpo MR1F | *hpo* | 5’CACCAGCTCATAAAAATGCCGTACG |
| hpo MR1R | *hpo* | 5’CCTCCGCCTCCGCCTCCGCCGCCTCCGCCTTGCGAGACGCTGCCCTCGCGATCC |
| hpo MR2F | *hpo* | 5’TGCTATACGAAGTTATGGATCCGAGCTCGGACCGAGGTAGCACTTCTCGAACAAC |
| hpo MR2R | *hpo* | 5’GATAGAGAGCCGCAAGGCTCAGGGAC |
