# Supplementary material for: Neurospora Importin α Is Required for Normal Heterochromatic Formation and DNA Methylation
Source: PLoS Genet. 2015 Mar 20;11(3):e1005083. doi: 10.1371/journal.pgen.1005083 (PMC4368784; doi:10.1371/journal.pgen.1005083)
Supplement: S1 Text — (DOCX) [file pgen.1005083.s021.docx]

**Detailed Materials and Methods**

**Nucleic Acid manipulations**

pBM61+*nup-6*^+^ for *dim-3* complementation was generated by amplifying WT genomic DNA with oligos 4286 and 4287, followed by cloning into the NotI/EcoRI sites in the *his-3* targeting vector pBM61 [67], for an ectopically placed, untagged *nup-6* gene. pP*_nup-6_*::*nup-6*^+^::3xFLAG, pP*_nup-6_*:: *nup-6^dim-3^*::3xFLAG, pP*_nup-6_*:: *nup-6*^+^::3xHA, pP*_nup-6_*:: *nup-6^dim-3^*::3xHA, pP*_nup-6_*:: *nup-6*^+^::GFP, and pP*_nup-6_*:: *nup-6^dim-3^*::GFP were generated by amplifying WT and *dim-3* genomic DNA with 4287 and 4294 and cloning the resultant product into the NotI and PacI sites of pCCG::10xGly::3xFLAG, pCCG::10xGly::GFP, and pCCG::10xGly::3xHA vectors [52], which removes the CCG overexpression promoter, and places the resultant *nup-6* gene that expresses C-terminal-tagged NUP-6 proteins under control of the *nup-6* promoter (1,000bp upstream) at the ectopic *his-3* locus. pCCG::*nup-6*^+^::GFP and pCCG::*nup-6*^dim-3^::GFP were generated by amplifying WT or *dim-3* genomic DNA with oligos 4293 and 4294 and cloning the resultant product into the XbaI and PacI sites of pCCG::10xGly::GFP [52], placing the *nup-6* gene (either the WT or *dim-3* allele) downstream of the CCG promoter for ectopic *his-3* targeting. pCCG::*dim-7*^+^::GFP was generated by amplifying WT genomic DNA with oligos 4346 and 4347, and cloning the resultant product into the BamHI and PacI sites of pCCG::10xGly::GFP [52], placing the *dim-7* gene downstream of the CCG promoter for ectopic *his-3* targeting. pCCG::*dim-7*^+^::mCherry was generated by amplifying the mCherry gene with oligos 4383 and 4384 from the pZero::mCherry::hph::LoxP vector (M. Freitag, Oregon State University), and cloning the resultant product into the PacI and EcoRI sites of pCCG::*dim-7*^+^::GFP (an identical cloning strategy, performed at the same time, was used to replace the GFP gene from pCCG::10xGly::GFP to generate the *his-3* targeting vector pCCG::10xGly::mCherry). pCCG::*nup-84^+^*::10xGly::mCherry was created by amplifying the *nup-84* open reading frame from wild type genomic DNA with oligos 4860 and 4861, and cloning the resultant product into the SpeI and PacI sites of pCCG::10xGly::mCherry. pBM61+*nup-6*^E396K^ was created through a Quikchange protocol using oligos 4342 and 4343 in a PCR reaction with pBM61+*nup-6*^+^. All clones were confirmed correct by Sanger sequencing.

All C-terminus targeting (hygromycin selection) cassettes were generated as described [52], except that a three-piece stitching PCR, using the forward primer specific to the 3’ gene end (to generate the initial 1,000bp C-terminal fragment) and the reverse primer specific to the 3’ UTR (to generate the initial 500bp UTR fragment), the generated 1,000bp 3’ end fragment of the target gene, the 500bp 3’ UTR fragments of the target gene, uncut p3xFLAG::hph::Lox (for example, other C-terminal tag vectors were substituted for the required tag; [[52], for the p3xHA::HA::hph::Lox and pGFP::hph:::Lox; Honda and Selker unpublished, for pDAM::hph::Lox; M.F. Freitag (Oregon State University) for pmCherry::hph::Lox], and LA taq (Takara) per manufacturer’s protocols, to generate the cassette, instead of the yeast recombination system. Forward and Reverse 3’ c-terminal oligos, as well as Forward and Reverse UTR oligos for *dim-7* (oligos 3642-3645), *dim-9* (oligos 3628-3631), *gcn-5* (4435-4438, integration confirmed with 4449), *taf-5* (4443-4446, integration confirmed with 4451), and *hpo* (hpoMR1F-2R, integration confirmed by Southern blot with an *hpo* probe made with a *hpo* fragment, below) given in Table S2.

The genotype of *dim-3* strains was determined by PCR amplification of the 3’ end of the *nup-6* gene (across the region containing the mutations) with oligos 4287 and 4307 followed by digestion with HpyCH4III (the R469H mutation of the *dim-3* allele abolishes a HpyCH4III site [wild type allele fragment sizes: 815bp, 712bp, 527bp, 50bp; *dim-3* allele fragment sizes: 1,527bp, 527bp, 50bp]).

For construction of the cassettes to reintroduce the *nup-6*^+^*::hph*, *nup-6*^R469H^*::hph*, and *nup-6*^dim-3^*::hph* into a WT strain, PCR was used with oligos 4307 and 4329 to amplify the terminal 1,000bp of the 3’ end of the *nup-6* gene, and 4309 and 4310 for 500bp of the *nup-6* 3’ UTR. All C-terminus targeting (hygromycin selection) cassettes were generated as described [52] using 4307 and 4310 for the in the three-piece stitching PCR, using the generated 3’ end fragments of *nup-6*, the 500bp 3’ UTR fragments, uncut p3xFLAG::hph::Lox, and LA taq (Takara) per manufacturer’s protocols, to generate the cassette, instead of the yeast recombination system. Note that oligo 4329 encodes a stop codon and bypasses the 3xFLAG tag, but includes the hygromycin cassette for positive selection. Construction of the *nup-6*^E396K^*::hph* cassette used the pEndo:: *nup-6*^E396K^ vector (above) instead of genomic DNA for generating the 3’ end of *nup-6* for the stitching reaction. Resultant stitching products were gel purified using a Qiagen Gel Extraction kit, per the manufacturer’s protocol, and transformed into Neurospora (below). Since the naturally occurring crossover events for stitching construct integration might have occurred prior to both mutations, in between the E396K and R469H mutations, or after the mutations (such that the mutations would not be integrated), mutations were confirmed by a combination of HpyCH4III digests (for initial genotyping) and Sanger sequencing (for WT, *dim-3*, and R469H alleles), or only Sanger sequencing (E396K allele). Correct integration of *nup-6*^+^*::hph*, *nup-6*^dim-3^*::hph*, *nup-6*^R469H^*::hph* alleles in individual Neurospora transformants were confirmed by PCR reactions of the *nup-6* gene with 4287 and 4307, giving a ~3,900bp sized fragment if the construct had integrated, or a ~2,100bp fragment if the construct had not integrated, and the fragments were digested with HpyCH4III, scoring for a fragment size increase, as the R469H mutation abolishes an HpyCH4III site (a WT product has a 815bp and 712bp fragment while a R469H mutation gives an 1527bp fragment, among others [above]); putative correct PCR products were Sanger sequenced for the E396K mutation. Correct integration of *nup-6*^E396K^*::hph*  (Figure 1C) in individual Neurospora transformants were confirmed by PCR reactions of the *nup-6* gene with oligo 4293 (templated at the *nup-6* 5’ end outside of the construct) and oligo 2354 (templated to the *hph* gene) followed by Sanger sequencing. Specifically in Figure 1C, the “*+R469H::hph*” strain was a crossover event occurring downstream of the E396K mutation in the *nup-6*^dim-3^*::hph* transformation and the “*+WT::hph*” strains was a crossover event occurring downstream of the E396K mutation in the *nup-6*^E396K^*::hph* transformation.

For construction of the *trp-2^-^::P_ccg_::dim-7^+^::mCherry::nat1* strains, a split marker strategy was employed. The upstream UTR of the *trp-2* gene was amplified with oligos 4878 and 4879 (containing 10bp of sequence that overlaps with P*_ccg_*), and the downstream UTR of the *trp-2* gene was amplified with oligos 4886 (containing 10bp of sequence that overlaps with *nat1*) and 4865; each UTR was amplified using wildtype genomic DNA. *P_ccg_::dim-7^+^::mCherry* was amplified with oligos 4880 (containing 10bp of sequence that overlaps with the *trp-2* upstream) and 4881 (containing 10bp of sequence that overlaps with *nat1*) from plasmid pCCG::dim-7^+^::mCherry. The *nat1* gene, conferring resistance to the antibiotic nourseothricin [68], was amplified from the pZero::3xFLAG::nat1 plasmid (Gessaman and Selker, unpublished) using oligos 4882 (containing 10bp of sequence that overlaps with mCherry) and 4885 (containing 10bp of sequence that overlaps with *trp-2* downstream). Fragments were gel extracted, and the *trp-2* upstream, *P_ccg_::dim-7^+^::mCherry*, and *nat1* fragments were amplified in a three-part stitching reaction (above) with 4883, which is templated 88bp from the stop codon of *nat1*. Simultaneously, the *trp-2* downstream and *nat1* fragments were amplified in a two-part stitching reaction (above) with 4884, which is templated 106bp downstream from the start codon of *nat1*. The two stitch products, which together have 355bp of overlapping sequence of the *nat1* gene, were gel extracted and together transformed into Neurospora strains expressing GFP constructs, per the hygromycin selection transformation protocol [24], except that plates with nourseothricin (25 μg/mL) were used for plating [68]. Presence of this construct at *trp-2* in transformants was confirmed by Southern blot probed with the *nat1* fragment (above, amplified with oligos 4882 and 4885) and with the *trp-2* upstream fragment (amplified from wild type genomic DNA with oligos 4866 and 4867). We note that strain N5740, while expressing NCA-1-GFP and DIM-7-mCherry, contains two *P_ccg_::dim-7::mCherry::nat1* constructs integrated back-to-back at the *trp-2* locus, which does not affect the interpretation of the DIM-7-mCherry localization in this control strain.

For analysis of nuclear transport with the NLS^SV40^-GFP reporter protein, we utilized a pCCG::NLS^SV40^::LexADBD::GFP reporter construct plasmid previously built in the Selker lab. To build pCCG::NLS^SV40^::LexADBD::GFP, the NLS^SV40^::LexADBD fragment was amplified from plasmid 3018 (LexA-d1EGFP, a generous gift from Dr. Hodaka Fujii, Osaka University) with oligos 3755 and 3756, and cloned into the AscI and XbaI sites of pCCG::N-3xFLAG to make pCCG-1N-3xFLAG::LexADB. Then, oligos 4958 and 4959 were used to amplify the NLS^SV40^::LexADBD to clone it into the XbaI and PacI sites of pCCG::10xGly::C-GFP vector, making pCCG:: NLS^SV40^::LexADBD::GFP, which encodes an SV40 monopartite Nuclear Localization Signal (N-terminus sequence: MAPPKKKRKVEPEG…; NLS^SV40^ sequence is underlined) fused to a LexA DNA binding Doman (DBD)-Green Fluorescent Protein (GFP) reporter construct for monitoring nuclear transport of the GFP protein targeted to the ectopic *his-3* locus. We note that the LexADBD does not actively bind any DNA sequence, as these strains do not contain the LexA operator.

**Neurospora Transformation**

To introduce the constructs into Neurospora, all ectopic targeting vectors were linearized with either NdeI, DraI, or DraIII, transformed into the appropriate histidine auxotrophic strains using a Bio Rad Gene Pulser at 600Ω, 1.5kV, and 25μFD, selecting for colonies grown on minimal Vogel’s medium plates, and made homokaryotic (or to place into a *dim-3* background) by crossing. Hygromycin selection transformations performed as described [24]. All putative transformants were checked for construct integration by either PCR using primers outside of the cassette, or by Southern blotting specific to the *hph* gene (for hygromycin resistance) or the *his-3* C-terminus.

To obtain the co-transformation strains N5819 and N5820, expressing NUP-6^+^-GFP or NUP-6*^dim-3^*-GFP respectively with the NUP-84-mCherry fusion construct, a 4,889bp NotI-ApaI fragment was excised from pCCG-nup-84+-10xGly-mCherry, as well as a 1,503bp BamHI-EcoRV fragment was excised from p3xFLAG::hph::LoxP. Both fragments, *P_ccg_::nup-84^+^-10xGly::mCherry* and *P_trpC_::hph* were gel purified and co-transformed into strains N4968 and N4970, selecting for hygromycin resistant colonies (above), followed by screening for mCherry signal.

**Southern blotting**

All Southern blotting done as described [54]. Probe DNA was amplified using T3 or T7 oligos specific to the Neurospora heterochromatic regions [20], or oligos 1877 and 1878 for the 8:A6 region or oligos 1864 and 1869 for the 8:G3 region. Euchromatic probe DNA was either amplified in PCR reactions (using oligos 3181 and 3182 for *pan-1*, or digested from plasmid DNA, as the *am* probe DNA was a 1.6Kb BamHI fragment from pMS2 [69], the *hpo* probe DNA was a 1.0Kb XbaI/PacI fragment from pCCG::hpo::3xFLAG (Rountree and Selker, unpublished), or the *hH3* probe DNA was a 1.2Kb HindIII/SacI fragment from pKA69 [61].

**Immunoprecipitation and Western Blotting**

Western blotting was performed as described [52] using the antibodies α-FLAG (mouse derived, Sigma #F3165; rabbit derived, Sigma #F7425; mouse derived HRP conjugated, Sigma #A8592), α-HA (Roche# 11 867 423 001), α-H3K9me3 (Active Motif #39162), α-hH3 (Millipore #06-755 or Abcam #ab1791) , and α-GFP (Abcam Ab290). Immunoprecipitation was performed essentially as described [52], except that isolated nuclei were lysed by gentle sonication in native lysis buffer (50mM Hepes pH7.5, 1mM EDTA, 150mM NaCl, 10% Glycerol) with 1% Triton X-100, cleared of nuclear membrane debris by centrifugation, and the resultant nucleoplasm was added to ~10uL of M2 FLAG affinity beads (Sigma #A2220), nutated overnight at 4°C, thrice washed with HC buffer (150mM Hepes pH7.5, 1mM EDTA, 250mM KCl, 10% Glycerol), and processed using standard western protocols with 8% acrylamide gels. Western blots were either developed on a Hope MicroMax developer using X-ray film (Phenix, F-BX810), scanned into digital images, and quantified using ImageJ (available at http://rsbweb.nih.gov/ij/), or imaged by the chemiluminescence setting on a Licor Odyssey Fc imager (http://www.licor.com/bio/products/imaging_systems/odyssey_fc/) and quantified using the Image Studio software (Licor). For quantification, all *dim-3* levels of IP bait or hH3 signal were normalized to the wild type level of IP bait or hH3 signal, and this adjusted bait/hH3 level was used to normalize the level of DCDC member-FLAG level or prey level in the *dim-3* strain. This adjusted level of the *dim-3* strain’s DCDC-FLAG or IP prey was divided by the level of the wild type strain DCDC-FLAG or wild type strain’s prey.

**Fluorescent Microscopy**

Strains for imaging were inoculated on slants and grown for four days at 32°C, at which time they were removed and allowed to mature for two more days at ~25°C on a light/dark cycle. Conidia were collected with a sterile stick and resuspended in 100uL of 50% glycerol + 1uL of 1:25 diluted Hoechst33342 (if DNA was to be observed; this ingredient was omitted if DNA was not to be imaged), and three microliters were spotted onto slides, covered with a cover slip, and imaged on an Axioplan fluorescent microscope (Carl Zeiss, Thornwood, NY) with a 100x objective lens and images captured with 200ms to 1000ms exposures using Axiovision software (Carl Zeiss). Z-stack images were captured with the same microscope and software, and Z-stack movies of Z-stacks were generated using the publically-available software ImageJ (available at http://rsbweb.nih.gov/ij/). This imaging protocol only visualizes paused, asynchronous cells.

**Isolation of Nuclei**

Conidia flasks were inoculated and grown for 7-10 days, and subsequently used to inoculate an overnight 500mL culture. Cultures were harvested by Buchner funnel filtration, washed with dH_2_O, weighed, and frozen in liquid Nitrogen (LN_2_). Frozen cultures were ground in a mortar and pestle kept cold with LN_2_, and resuspended with an equal volume (weight:volume) of Buffer A (1M sorbitol, 7% Ficoll, 20% Glycerol, 5mM Magnesium Acetate [MgAc_2_], 3mM CaCl_2_, 50mM Tris HCl pH7.5). Culture slurry was filtered through a cheesecloth funnel prewet with Buffer A. Buffer B (10% Glycerol, 5mM MgAc_2_, 25mM Tris-HCl pH7.5) was added (2x the volume of buffer A) to the supernatant. Following overlaying of the solution on additional Buffer A:Buffer B solution (ratio 2.5:4), the supernatant containing cytoplasm and nuclei was centrifuged at 3000xg for 7 minutes in an HB-4 swinging bucket rotor at 4^o^C to pellet cell debris. Supernatant was overlaid onto ~1/7 total volume Buffer D (1M Sucrose, 10% Glycerol, 5mM MgAc_2_, 25mM Tris-HCl pH7.5), and centrifuged at 9400xg for 15 minutes in an HB-4 rotor. Supernatant was discarded and the pelleted nuclei were resuspended in Nuclei Storage Buffer (25% Glycerol, 5mM MgAc_2_, 0.1mM EDTA, 3mM DTT, 25mM Tris-HCl pH 7.5), flash frozen in LN_2_, and stored at -80°C for use in subsequent experiments.

**Additional References**

67. Margolin BS, Freitag M, Selker EU (1997) Improved plasmids for gene targeting at the *his-3* locus of *Neurospora crassa* by electroporation. Fungal Genetics Newsletters 44: 34-36.

68. Kuck U, Hoff B (2006) Application of the nourseothricin acetyltransferase gene (*nat1*) as dominant marker for the transformation of filamentous fungi. Fungal Genetics Newsletters 53: 9-11.

69. Singer MJ, Marcotte BA, Selker EU (1995) DNA methylation associated with repeat-induced point mutation in *Neurospora crassa*. Mol Cell Biol 15: 5586-5597.
